# Supplementary material for: Using Theory of Change to inform the design of the HIV+D intervention for integrating the management of depression in routine HIV care in Uganda
Source: PLoS One. 2021 Nov 30;16(11):e0259425. doi: 10.1371/journal.pone.0259425 (PMC8631669; doi:10.1371/journal.pone.0259425)
Supplement: S1 File — (DOCX) [file pone.0259425.s002.docx]

**Preliminary ToC 1 in Mpigi**

Participants:

- Administrators

- Health managers

- Religious leaders

Code

F1 (facilitator 1)

F2 (facilitator 2)

PI (Principal Investigator)

MoH (Ministry of Health representative)

R (respondents/participants)

F1 It is going to be very simple and I want us to take an approach that will make us comfortable. The first time I attended or I heard of Theory of Change….it seemed very complicated. When I was a Masters student at Makerere, we had a professor….see, now I do research, specifically qualitative research; but we had this proud Professor….whenever he came to teach us, he would brag a lot, talk about his projector (by then he would tell us it was 10 million shilings). He used to call it “10 million technology”. He was driving a Benz. He used to talk about how expensive his Benz was, how he had very expensive contracts/deals. Then he started teaching research methodology, using complicated terms; Rural Rapid Appraisal, etc. Many of us got frustrated and almost lost interest because of the language he used. He was always bragging… we really lost interest. We really struggled because we knew we had to pass anyway, but he frustrated us. Later, when I got into research business, I realized things can be made simple if we choose to. His 10 million technology and Benz are no more, but he is still around. The other time we met in Butabika and he needed my assistance and that colleague of mine. We couldn’t avoid remembering and talking about the way he almost complicated life for us when teaching. The message am putting forward is that it’s not proper in the teaching profession to complicate life. But am not here to lecture; am not going to handle this as if we are in a classroom environment. I simply want us to appreciate this …as professor has taken you through, we are trying to adopt an intervention which has been developed and implemented in India. I think those days India was regarded as the developing country. Now it has moved to a middle income country; but some of the states in India are still very poor. The MANAS project he is talking about was developed there tested and was successful. It was all about using lay health workers in integrating an intervention in the PHC setting. As you know the health systems in most of our developing countries, we barely have adequate specialists yet some of the conditions that people present with may require specialists. When we get to the PHC where problems are presented, we don’t have specialists at that level. So, in most cases problems go undetected and therefore untreated. That’s the reality. Now take an example of the problems we are talking about today- depression in people with HIV, sincerely…..maybe what professor did not tell you is that he earlier conducted some studies which found that depression is very common among persons with HIV. He did not just wake up one day and said that- let’s have depression management into HIV-care. The other day we were launching this program, I shared with people my experience when I once worked with an NGO which was not successful. I was the program officer, working with Japanese. We were providing psychosocial support to AIDS orphans. Which sounded fine. But with time, as we implemented, I realized that my bosses didn’t even know what psychosocial support is. So what they were trying to do was some crude form of counseling. You counsel an AIDS orphan for a year, two years…..three years…. you are counseling the same… orphans. One who came on board while in P.2 gets to P.7, does PLE, goes to secondary…..you still claim you are counseling. You know!…. I remember telling them that the program was not based on realistic needs but rather, assumed needs. It didn’t go well with them and eventually, I had to leave. And that often happens. Some of us have been involved in such projects….even research projects for which the Investigators just think of the theme… maybe after doing a literature search. Sometimes they just think of the interventions. Some of us have been involved in such projects. You go to a district, you either take the intervention, you just say we did research … we found this and this, …now we are going to do this and that. We’re going to train health workers, we are going to do this, we are going to setup this- we are the experts. . So that way, we are imposing things. In this project, we are going to take a different approach. We are not imposing interventions. We have come to you before the intervention and we need your ideas, your input…and we shall say we worked together to develop the interventions.. Remember there should be buy-in as he said. We have worked in some districts and uh it’s good… Mr. Tarsi is here, Dr. Sheila would often…uh… there are districts we would go and talk of the mental health policy, and…. I don’t know whether you are aware that most managers don’t appreciate the difference between a policy and policy document. You ask them about the health policy, they will say the health policy is somewhere (then he looks up for the document) or the strategic plan – they say “uh we have that plan…its somewhere in DHO’s office”. Even for the interventions we are developing, we don’t want to have a write-up that will end up lying in the DHO’s office in shelves, with a lot of dust. We are developing it together so that you own it… so that we minimize implementing mistakes as we said earlier. And that is what Theory of Change approach is about. We are using it to adapt the MANAS intervention which was successful in India. So we are adapting it here. The other was addressing common mental disorders, specifically depression and anxiety disorders. So again we are talking of depression the common mental disorders. Common in the sense that they are very common at OPD … in the PHC setting; where they mostly go undetected and untreated. As we adapt it, we are working with you to identify those interventions that are feasible, practical, and realistic in our settings.

Mr. Kaggwa…. I thought that among those invited, we have some facility managers. We have tried to buy time, but we’re not seeing them. It will be unfortunate if they come in towards the end. They will not contribute much..

So, in this Theory of Change workshop, we are not going to complicate life like the other Professor. We are going to be active participants, not passive recipients. Let me begin by giving an example of one project we worked. We did some research, we conducted a situation analysis of the health system in a given district, we analyzed data, and we planned several interventions based on the findings. We went to this district and we told them that after our situation analysis this is what we found….and these are the interventions we are proposing: training the health workers, training the village health team members in mental health care, setting up a multi-sectoral forum for mental health in this district, forming user support groups etc. We listed the interventions and the DHO of this district and his colleagues did not object to whatever we proposed and we had the resources. So we went ahead and implemented most of these interventions as we had planned. And there was an evaluation phase. Unfortunately, we are the ones who did the evaluation again. But after the evaluation and the closure of the project. I went back to the district once in a while to check, but found almost nothing. Things had ended with us. Even the MH service users whom we had given some piglets and bla..bla….. as part of the income generating activities strategy had sold the pigs…(LAUGHS). So, there was nothing. Traditionally that’s what happens……we do research and plan interventions… we just go in and do the interventions. Unlike this approach, using ToC does things differently. We undertook the interventions wanting to achieve something. We trained health workers without knowing whether the health workers were interested or not. At planning stage, we didn’t even know whether they existed. We simply moved in with our interventions, with ultimate goal of seeing to it that mental health is integrated into PHC in that district. So that is the traditional way of doing things. With Theory of Change approach, we do things in a slightly different way. We want to see change, but for any change to occur, there must be certain things that come about for that change to be realized. There must some underlying theory…..there must be some underlying theory… am just trying to simplify things. What this approach does is that it involves stakeholders in a very participatory manner, you determine where you want to get…..are we together…… we determine the change we want to see or the impact we want and then we work backwards. We are saying here that traditionally managers would develop their plan either based on assumed needs without even having done any research or they might use some research findings… then they sit and plan for the interventions to undertake. So they will put together these interventions which are expected to bring about the desired change. And the hope that the interventions will be undertaken but without any clear understanding of why they are doing what they are doing. For example in that other project, we did not know why and whether income generating activities was the best option. Some other organization had been doing that in some other districts….users are given chicken or piglets and we said “we should have some interventions with the users- those other organizations have been doing it – I think we should also do the same”. At this level what should we do? But why are we doing it and what do we expect. And is that the best way we should do it. So, the why? The how? are usually not explained or understood well. So with the theory of change approach we do things the other way round. We identify the desired outcome and we work backwards to identify interventions and strategies. There are two key things in this- the desired outcome or impact and the interventions or the activities. But we do not start by identifying the activities as is usually done. Like we would say “ lets go to Mpigi, train health workers in mental health care, emphasis depression, tell them that they should screen, do this and that….so we determine the activities which we think will lead to the desired outcome. Here we are doing things differently. We are determining the desired outcome and work backwards, identifying the preconditions that have to be in place for the desired outcome to be realized. I hope we are together. So we are going to determine what we want to achieve in this HIV +D in Mpigi and beyond. But for us to get there, certain things have to unfold. There are certain outcomes we have to realize… some are short-term some are long-term. There are some activities that have to be undertaken. We don’t know which activities they are. There are some indicators – there are things that will show us that this is happening, that here we are on track. We have some assumptions to make. We may plan things based on certain assumptions- or we think the system is fine, we think it is acceptable or we think the health workers are there. We think the health workers know what depression is and we are basing on wrong assumptions. So we have to clarify those assumptions…… that for this to happen, this must be there. Once we have this happen then we shall know that this will happen. But for this to happen, this and this must be there, E.g the medicine must be there, the health workers must be regular, they must not be dodging. We have seen some facilities that are run by community health workers- VHTs. We have worked in some districts where the in-charge appears at the end of the month only….where the Records Officer appears at the end of the month just to prepare the HMIS report…. and he gets figures from the head, not from the clinical registers. At any time you come to the facility you will find a community health worker and maybe with a nursing assistant always. But if you invite them for a workshop they will always come. And you teach them…. They will be very happy and excited. You go back to the facility you find the materials you gave them scattered in the corridors. These are things some of us have seen. So you invited them on the assumptions that they are willing to learn and change something. And you have the assumption that work goes on as usual. Not knowing that things are upside down. In some district I reported to the DHO, I first talked to the biostatistician- told him the information you have is different from what these people have at the health facilities – I showed him the difference – he seem not interested. I showed him this is what you have for this health facility for June. When I went to the health facility I find a different thing. So who is telling lies.. He didn’t …. And when I talked to the DHO, the DHO – unfortunately said ….ah that biostatistician ….he is apparently more powerful – even CAO cannot summon him, because (laughs) because he has a God-father- we usually say godfather. But this one was a Godmother… a powerful politician in the region. So he comes around when he feels like. Not even the CAO can touch him. So there might be such challenges – that’s why we talked of …as we plan the intervention.. the feasibility ..what are the likely problems and what are the solutions in case those problems are there. Will Mpigi be like that other district where the health workers will not listen to the DHO, if she tells them no. these are things that we will come to identify as we plan interventions or the activities. Once we have identified all those – I think we shall be moving back and forth. Graphically ……..(draws a schematic on flip chart)…..what this looks like. As you see there we are starting with the long-term vision. We may call it long-term vision or impact. What we want to see. We are going to start at that level and then we work backwards. That’s why you are seeing the arrows- the arrows at least are moving backwards. So we determine the impact we want to have, we determine the outcomes and then the results, the activities, the assumptions. All those have to come in between as we move backwards. Now when we identify all these and put them together, we have to put them together in a way that flows logically and makes sense. So we are going to begin by identifying our impact or the long-term outcome- we might have or could call this the impact….if we choose to … we may call I the long term outcome. Depending on the ideas we get from you- we could have several other outcomes along there and then some other short term outcomes, then we work backwards again to determine the activities to be undertaken for us to achieve those outcomes. But in determining the activities, there other things that come into play. We have some assumptions that we are going to make. We have to identify which assumptions we are going to make. Then indicators, what are the things that will show us that this is happening and so on. We have to map them.. to connect them logically – we have to connect-to see how they are interrelated . We have to identify who the key players are. The implementers.. who will be doing this, who will be responsible for this. We are talking of say the counselors… are they there? If they are not there, who is going to serve as the counselor? So we identify the implementers and who will be the players. And then the resources finally. We shall put this together in a way that flows and makes sense and what we shall have created is a theory of change map. And that theory of change is a very nice summary of interventions. It can be used as a planning tool for whatever is going to be done and we can also or we shall use it for evaluation because the indicators are already identified.. so it makes work very simple for us if it is well done. But for this preliminary workshop, this is the initial one – we are going to brainstorm, when we go back we shall put it together as a team. Next week when we come to meet other people. We shall get ideas from them. Then we shall have a task of putting them together, then we come back we shall look at the theory of change map we shall have got. And say that for this program – this is how we are going to go- these are the outcomes we expect and this is how they are going to unfold… this will happen but before it is there this must have happened- before that one this must have happened- these activities must have been undertaken, these are the indicators that will show that things happened. Once we agree on that then the intervention will be rolled out. I don’t know whether I am clear. I think I have tried to make it simple and understandable. Now let’s get to business

Later on we might have to use these things. You remember the introduction. Let’s start by identifying the impact. The impact might be …. We were using the term vision. I think vision is sometimes something that we might not see… we may not live to see. But we are almost trying to get to it. Thought we may not realize it especially in the life of the project. So usually what we do... sometimes we put there what we call ceiling for accountability. We may not be accountable or very responsible for not achieving that but at least we tried to achieve it,

For this meeting we are not going to draw the map-I am just demonstrating- it will be the technical team to put these ideas together in the map. But at least I want to first demonstrate. We are determining the impact… we can even extract it from the proposal. We are talking of depression managed in HIV care, or management of depression in HIV care… that is what the project is all about…we can phrase it differently but we have the impact . and as we said it maybe something that we may not achieve fully.. now this broken line is like a ceiling that we may not be held responsible or accountable for these long-term outcomes. And we know these long-term outcomes should eventually lead to that impact once they are there. So the project might end before we are fully there but once these are there, then we know we shall achieve the other. Am using LTO for long term outcome and STO for short term outcomes. Now some of the short term outcomes might also feature as long term outcomes. It’s not a must that we must identify short term, long term, intermediate, some of them may not be … so the term to use is outcomes and we shall just identify the outcomes. During the time of developing the map we shall see whether some outcome is realized immediately after the activity..so we come here and We do an activity and within two days we expect….. that is definitely a short term outcome. There are some outcomes that we shall expect some time after … say change in attitude or the help seeking …so I do know how to mention… so we shall just be talking of outcome and we shall see where to put it in… so outcomes, these are the outcomes… am using a triangle for indicators and we are using AS for assumptions. we might say this activity is going to lead to this outcome but the assumptions would be between the activity and here …. For example you are training people and you think that once you train them- they are going to have the knowledge but you are assuming they are interested in the training. You might invite 20 people and you get three, because they are not interested. So the assumption was that …. I don’t know whether you are getting me … so we would have worked backwards to get to … so we are not starting with the activities. And probably my colleagues are [not clear ] we have not sat and said lets go to Mpigi and do this and this and once we do that, depression will be integrated in HIV care. Can we start?

P Can we start at the activities and get to the impact at the extreme end?.

R Oh ok… you were out when I explained this.. I said that traditionally when we are planning we start from here . You sit in your office, you plan… you want to achieve something. You want to have your health workers active and then you say ok… lets introduce some allowance... every end of month we give this much. You have not thought about… have you thought about that incentive you are introducing, how it will work, why you think it will work, how you have to operationalize it. And so on... so you sit you plan, you are looking at what you want to achieve. Think of what you have to do and you do that and expect to get there. As we have said many times we do not get there, because we have not identified the assumptions- which might be wrong. We have not identified the indicators which might show us that we are not on the right tract maybe we change. So that is the traditional way of doing it. And we were saying for us to avoid implementing mistakes – because when you start here and do things – you realize that you have made mistakes when it is too late. Now we are trying to understand the entire theory behind the change that we want to see by identifying all these components which are surrounding the interventions and the change that we want to see. We identify them in advance and once we can have them in a way that flows logically and makes sense, then you go out to implement and we shall be implementing this and at any point you shall know that you undertook this activity- the other assumption and this assumptions were catered for. So what is the indicator, what will show us that people are working. You will have already identified that indicator. So if you are doing the support supervision or monitoring visits…. Madam DHO, I don’t know the monitoring and support supervision in your district… I have been to some districts where you talk of monitoring and support supervision- what they call support supervision is paying a visit to the health facility- when someone more senior pays a visit---you know- how are you… bla—bla some five - ten minutes … then that is recorded as support supervision. That they are doing support supervision, the monitoring.. it’s very interesting but… it’s true that some time we don’t know what we should look out for when we visit the health facilities. So if we have identified the indicators as well, that you know we shall be getting data on this, it will add impact when we see this- when we see this, say when we see that number raising to this much, or when we receive such a comment… then we shall know [not clear] so when you are going for monitoring and evaluation you will even rely on the indicators that you have already identified. So it will become easier. Do we all appreciate this.. is there any question.

P I don’t know whether it is a concern. But in your beginning, you talked about identifying the barriers and then eventually we come up with the strategies – you drew that picture am seeing you as someone who has stopped at the strategies. What about the “why?”, the why question. How are we answering it. Like we can ask ourselves what brings depression..

F1 Are you giving an example?

P Am giving an example that you have received a patient that is having this. He is depressed and you ask yourself “why is it like that?”. Ok everybody might say it is because of HIV but there are other issues that contribute to that incident. So as you are giving us that picture, I am seeing you as someone who has stopped at the strategies..

F1 Stopped at strategies?

P Something like activities. ok strategies that is something you can come up with activities… then you ask yourself- I have treated this someone but what brought about this thing or what brought about this depression.

F1 O.k, I get you. But for now, we are not discussing the actual management of depression,…the actual management of individual patients of depression. Today we are looking at …how can we bring this management, this service, this screening. How can we introduce it into HIV care. Today we are looking at the service, the health system, how can we change the health system to make sure that depression management is taking place. We are not looking at individual patients. That’s the difference. This is a health systems approach that we are looking at. Sister that question, I think… I don’t know whether you will be part of the other activities. That would be a very good question if one is training the lay counselors or the health workers who will be handling the psychotherapy. Huh that when you identify, you screen and identify this person as having depression- then you have to assess and understand where did the depression come from, what could have caused it. Is it just mere fear of HIV, is it the stigma, is it … when you are probing such issues- there you are looking at the management of the individual – the condition [not clear], so this is for the program, for the service and we definitely….

F2 And I think Joshua, her question is really good. We need to assure her that for the different groups that we are going to meet, different diagrams will emerge. And we eventually put them together. And finally come up with one that will be representative of exactly what we want to focus on. And the technical team will put it [not clear] a guide that will keep on growing. Only that as professor has said that we are looking at the broader service delivery system. But when we meet other groups that will be part of the focus and the diagram will keep on growing and growing

F1 This is still an example. We shall go into practical tasks, so this is just for demonstration

P Just something maybe we need to get clear…now that all those levels… we have at the extreme end- impact, which we really are very optimistic about and here at the lowest level we have actions what do we mean?

F1 Yes

P O.k, action points. So ideally, where should we begin?

F1 Yes. Now the actions… that’s where we are getting. As I said, we are beginning from not from left to right as usual, but from right toleft .

R I was relating it to an intervention, for example in a way of addressing depression- family counseling. Now if a look into those levels, where do I put it? Because I see you are telling us that we have to work retrospectively.

F1 Very good, what is happening here… as we said, this is a…..it’s a research workshop, that’s why we are taking note of all your suggestions and ideas. Because we are not going to implement pre-determined interventions. Otherwise… so we want to collect as much as possible…that’s why I said… you are not going to be passive recipients, in any case we should be the ones now receiving. So we are getting as much as possible from you, and then we shall try to synthesize what we have –put it together and then we come-up with the interventions we have to undertake and ….but again we shall have to have consensus after getting all these ideas, it’s like brainstorming. Yes we may know ideally what we may come and do in the district, but then there will be no sense of ownership to come and say there are these interventions- do this …do this. Get counselors lets train them and walk out. And there will be no empowerment or capacity building in that case.. so this process is going to help us appreciate this because we work together so that we don’t just impose. We could sit... Through our books, experience and so on and we decide, but it’s not what theory of change recommends.

R Probably it would have out clearly if we had used a real example. Because we know our impact. We want depression identified and properly managed

PI That is the next. That is what he is going into now. He wanted to just give you the picture and now he is going into that. That’s why we have called this meeting. We are going to leave theorizing about past experiences, we are now going to see –how do we operationalize depression management. So please get in, everybody is eager to go there. Take us there now.

F Unfortunately, I don’t know whether…..am looking at the width of the flip chart stand. I would have loved it on landscape [arrangements for recording the tree on the ground]

R Should we stand around you?

F1 Not yet….

F2 We do have a lot of time… as I said after this and the next preliminary ToC , we shall have to put all these things together. Then in the final Theory of Change workshop we shall have come up with the theory of change which we shall be discussing for consent. We cannot draw the theory up here in this preliminary workshop. This is going to be for demonstration, to have an idea- the ideas you are bringing, where are they falling. But we shall do it better, then we comeback with you and others. Where are now as you can see the other slide. You are seeing stakeholders brainstorming, discussing [not clear] we have been talking about something similar and we are saying that will help you avoid implementing …..just as simple as that.

F1 I wish all planning approaches were taking that approach- otherwise we have districts where we have the chairman, CAO and a few people deciding for everyone. Certainly you see a bias- because of their background, religious, occupation or political interest and so on. But [not clear] we have plans that are relevant, easy to implement and sensible for the reason that you will have understood the reasons, the why and how of those …….in …ideally -it’s not that we are going to come up with activities or interventions that are very complicated. They may all be the same usual activities, but the fundamental difference here is that having understood why you are undertaking every given intervention and how we should do it. For example one time when we were planning the training we had to debate- these health workers- should we invite them to a hotel in Kampala? Should we invite in this district. Some were suggesting that these health workers should be at the facility, and as you train-how are you going to train them or how are you going to teach? One may think of lecturing. Another may demonstrate, explain and make life easy. So we are into business now- we are beginning by- now let’s forget about Uganda, now we are in Mpigi though the program is integrating of management of depression in HIV care in Uganda. So what should we state as our impact or long term goal? or we can just air lift it as it is in the proposal and put it here or we can refine the statement a bit. How should we state it?

R We add the word “routine”

F1 You are saying…where should we add the word routine?

R Routine management of depression in HIV care

R Yes, routine management of depression in HIV care

F1 Why are you adding the word routine, why routine management of depression. What happens if it is management of depression? How are the two different? Should the management of depression be routine.

R I think it has not been about the quantity, it should be effective

F1 Aha, someone is bringing another word- effective. Effective management of depression in HIV care.

R Patients assessed for depression.

F1 now you brought in another word -assessed – we shall have reasons why we are having all these words -we cannot manage depression which is not assessed

R We are just emphasizing

R That routine is already a fact which we may not reach in the life of the project

R management- we must manage depression but what comes after –quality of life

R We want to promote wellbeing because what we have now is quality of life. So one of the things, it takes away their mental wellbeing... because when you manage depression you have managed anxiety as well because we are looking at the common mental disorders. we want to promote mental wellbeing.

F1 but what will be the impact after managing this

R you want something broader?

F1 yes

R bigger than depression

R because we also have to manage non-communicable diseases, we have to manage hypertension

P I think it is that one, the other ones look like either long term goals

F1 for now we can agree that we can put this

R to promote mental wellbeing

F2 the impact should be felt in the community

F1 So, DHO you are saying “management of depression integrated in routine HIV care” can be one of the long term outcomes

R now for the side of the sociologists – we need to promote reduced violence among families of people living with HIV

F2 That one looks like it’s among the outcomes

F1 Like I said some will come as long term outcomes, others will come as intermediate outcomes or short term outcomes, but for now we welcome everything. What were you saying?

R Reduced violence among HIV families living with HIV

F1 Any other? .. maybe at this stage we can set… we are looking at impact or outcome.. ok we said we want to promote mental wellbeing of people living with HIV/AIDS. For people living with HIV/AIDS to have their mental wellbeing –what must have happened? What must we achieve for us to be there?

R We must first remove the depression, one of the causes of poor wellbeing is depression- so we need to remove the depression.

F1 so how do we remove the depression – how do we state that, that is an outcome- how do we phrase that outcome?

R integrating

F1 now integrating is one thing and removing depression is another thing- we may integrate the care and the depression remains,

R integration into management!

R you are asking something between management and the promoting. Is there anything between?

R what we are saying is that depression causes poor mental wellbeing, so we want to remove it. What activity is there? What should we do to remove it? They are saying we integrate depression management into routine HIV care

R ok, maybe if I may ask – how shall we know that we have integrated. How are we going to integrate and how shall we know that we have integrated

R assessment for depression

F1 aha now Madam DHO you have jumped.

R but we can keep writing- we shall forget it.

R so integration involves what? –it involves recognizing it, which is assessment or screening for it. And giving the appropriate treatment. So we need to integrate depression management but how are we going to integrate

F1 that’s where we are going

R so suggest how are we doing that?

F2 the how are the activities

R We are talking of assessment. We need the health workers to understand the signs and symptoms of depression so as to be able to identify those patients easily. Otherwise, they will keep missing. As you know, in our culture, there is no word like depression.

R like to empower them to diagnosing depression

R Yah to build their capacity to diagnose

R they have to first appreciate the mental health issues in HIV

R so we have to build their awareness.

F1 Only health workers, what about people like Betty (expert client), can’t Betty see that a person has a problem. And say that this is depressed.

[not clear]

R recognition –you need to recognize the signs and symptoms then after you recognize you should be able to handle.

R but he is bringing up an issue before that

R and appreciation that is something to do with attitude.

R yah

R The how to and the what to in terms of knowledge, attitude but also the practices of the people who are doing HIV management- that’s why I think that recognition part of … that is attitude ..for them to first feel like… recognizing that it is a problem and can be addressed, and by them. So that is attitude. Then the knowledge …they know that ….. are we able to identify, screen, identify document, intervene, refer, follow up. All those are part of knowledge but also practice. So do you have anything on practice…practice knowledge. But also… because we have systems and processes. Most of these might be placed anywhere. But also we have the system now –the health system itself- which involved the infrastructure and those other things- logistics …yes…so the individual… the individual health worker is part of the complex health system. But we have all those other things. So what do we want to see? Eh what do we want to see in terms of the health systems? By and large we need a system that will support what we have said the removal of depression. How do we want that system to look like-that system that will support the removal of.. . we need a system that will support those processes. We need the medicines, infrastructure in place, tools and job aids—that complex thing. It can be broken down, am sorry I hope am not complicating things. We need to look at both the systems the processes—process is about how do I do it? —steps. A lot of the time we know the “what to” – in terms of the checklists. If you have forgotten this is what you should do. But we fail at the “how to”. That’s why we are saying you have got to face the [not clear] documents –guidelines and policies. They are there. The content is there- a lot of it. But getting this content now and making it practice. I have been to facilities for example in HIV testing- there are all the SOPs, but someone is still doing it as they[not clear]. So having the content, the what to is one but the how to is as well very important- that’s where now all the processes work together- structure, the referral systems, linkages and referral, the community, our PLHIV networks all those are now part of the complex process. So what do we want to do at each of those levels for us to be able to remove depression? I think that might form part of the introduction. Systems, human resource – which has already been mentioned- human resource.

F1 human resource- that is an assumption…because we are doing more as we are brainstorming. If we agreed on the impact outcomes – we would just talk of the outcomes , some maybe long term, some maybe short term. Some of what you are saying will come as assumptions later as we complete them backwards. So we have one outcome- we have management of depression- it will later lead to our impact of promoting the wellbeing.

R So in terms of outcome, that integrated management of depression can it be broken down now into the knowledge, the attitudes and the skills of the provider for example. Because for me I would like to see

F1 Aha you are talking of health workers having…o.k, so now you want to break down the integration – lets break it down…that’s a good idea..

R Breaking down that integration will now bring us to what we want to do to achieve those things

R That’s what I was whispering – what are those major changes that must be constituted to see there.. that’s the integration?

R The changes we need to do – we need to remove the depression, how are we removing the depression- by integrating management of depression… now he wants us to go backwards.

F1 Yah. How are we integrating depression management? What does integrating entail?

R That reduces the abstraction, and then we get another strategy – that for us to see that we also do this and then we move backwards- what he is suggesting is what will loosen the whole thing.

F1 Ok for now that is the major outcome –what do we have- what does it entail- at health facility level, at health organization level, health management level at community level and think of those changes- what are the changes that we will see?

R The evidence of integration, are the things now that we need- then will lead us to what are the things that we must do- then that will lead us to what we need to do to those things that lead us to that .

F1 now we are speaking the same language. So what are the changes that we must see? Should we start with the health management or health organization level? The DHO, DMT…

R I think we can start small with the service providers, well also the community, but that whole web of health systems that I thought- that system the is helping us to achieve that, that is responsible, that is willing- managing depression in HIV. So what are those things? Had mentioned like for the provider all the three things the knowledge, the attitudes, then the skills or practices.

F1 health workers- health providers knowledgeable, with positive attitude… that will be an assumption now.

R Because we know that even mental health workers have stigma for mental health. Even the health worker- mental health worker for us- we also have stigma for mental health.. they get someone with depression! They say nothing –these ones should go …these are the health provider

R You talk of the infrastructure

F1 The health provider… how about now the health facility; what changes do we want to see at health facility level?

R Wou want to see antidepressants, wou want to see medications there, available medications

F1 It seems you have a point.

R On health providers’ level, are you going to get people to train- to be trained at the facility to give that service. That is the knowledge and skills

R She should be suggesting…

R Of course when we talk of the knowledge and skills- that implies the need for training as an activity. Because if there is a knowledge gap, that suggests need for training. Available medications –maybe another

R I don’t know whether am lost.. If am a patient, I will want go where I can find drugs. So I want it to be a functional health facility- where one can find all the drugs, the health workers, the health providers and other things.

F2 We want to focus on depression, so that’s why we say available medications for depression, we also want to see available psychotherapists

R I think what she is saying. If that facility is functional. What should that facility have so that it can be able to provide the services?

F1 That is some outcomes and some assumption- so indicators. So what should we expect of a serious health facility?

R I want to see a facility that is free of stigma even when people come and have their mental health issues such as depression…what do we write there- free from stigma? And then we can move backwards to identify what needs to be done at that facility.

R Yah, one where people can freely open up with their mental health problems…where people don’t feel stigmatized.

F1 HIV stigma or depression?

R No depression- the mental health… because a lot of time the people shy away. But also the facility or the system.

[not clear]

R Once they are not there…sometimes we forget

F1 What are you saying?

R When the patients come into the health facility… is there a chance of capturing data is there a specialized form - kind of a gap- where a medical person can record.

R We have been having an issue of screening and actually the guidelines talk of – there are two questions.. there is something called the PHQ-2, where there are just two questions. One question is –have been feeling sad, then have you lost interest in formally pleasurable activities. So you are talking about screening.

F1 His concern was about the records- the screening tools

R The HMIS… but HMIS is already captured.

R Which is that one you already mentioned

R PHQ screening- if someone has one – you refer him for more detailed assessment- that is where you will have the PHQ-9- which are now nine questions

P A number of staffs who will be involved in addressing the issues of depression at the facility – is it the entire staff or some..

F1 I think that is the question….. What is the issue?

R That the number of staffs at the facility –those ones to handle people with depression has also to be looked at as an impact. Is it going to be a limited number or the general population or general staffing?

R It’s like if there is somebody with depression- at least everybody should be able to identify –even if you are dealing with somebody and you should be able - Every health worker should have the knowledge to quickly administer these two questions everybody should be able to recognize and refer. And then refer for more assessment..

P Right from the triage.

R Because the depressed person may appear anywhere- she may even have come for milk or some donation or something. The person picking this donation should be able to pick the symptoms. And refer for further assessment of this problem.

R It would be nice to also do follow up.

R Maybe follow up is a big thing- because follow up happens..especially when we refer them to another point of care.- what happens. That’s usually the gap.

R Referral to mental health-like actually we said you may find that the problems is domestic violence at home so you can’t refer to ….[not clear]

P but also there is the community-facility referral. Now we are talking about the queues. I don’t know maybe in 20…….the people at the community level....Ok… I want to see our community based resource persons such as the PHA networks being able to identify some of those issues like depression and linking the person to the facility. The community –facility referral

F1 Which I think should be the work of the VHTs ideally, because all of them ….[not clear]

R Yeah, whatever structure is there. There should be that component in their work.

F1 So, what needs to be done for us to have that? So you also want referral from the community?

MoH Yes, community-facility, because we have some of them who are actively involved in follow up of people living with HIV- yeah. They follow them up [not clear], now we are going to start DSD- differentiated service delivery- where some people will even be picking ARVs in the community.

R differentiated what?

MoH Differentiated service delivery for HIV care. Some will having representatives come to the facility coming and picking drugs for like a group and take for them. So in that group down there for example – how do we integrate these. What are those things that the group leader …actually in some districts.. someone was saying in districts like Bundinbugyo- they were looking for someone who can read and write among the group but they could not even get one- but this group leader for example of about 15 people living with HIV- who have this community lead ART group. Yah. What can that person do in that group? One, two three things.. I just want it to cascade right from the national referral up to the community. All those levels. CLAD is a very…. actually right now we have a training of trainers in Jinja for national trainers who are going to be rolling out the differentiated service delivery. I will share the guidelines with this team. And then you can see… the implementation guidelines… is there anywhere where we can put something. Because that is going to be the way to go. We want to decongest the ART clinics. Patients who are stable- be at the community other will be coming to the facility but they will only reach the triage desk and go pick medicines from the pharmacy. Yeah.. so at that point for example we will need something at the triage desk. Somebody manning the triage desk -can they be able to identify a depressed person. Who is a person living with HIV, who is a lay person. So those simple things for that lay person to be able to pick, identify a red flag and say *um omuntu wange ono alabika tali bulungi.* Because ideally we are saying these people are stable but …..laughs…..this stability is not permanent. Its not permanent especially when you have a chronic condition like HIV. Yeah today you are having issues…...you are not adhering the other day you are stressed –stress could lead to something so that’s why having it done everywhere… one or two indicators everywhere.. at each level of care will be very important.. The work is becoming too much [laughs].

R Ooh! we are getting the things.. you are saying differentiated service delivery – you also want it integrated there. At the triage desk at the health facility there must be somebody who can raise a redflag at the health facility where the …. as the patient is identified, are there forms where ….we can capture that kind of information?

R I think in the HMIS there is a depression code. There is a depression code in HMIS but maybe what is missing at screening, because when I compare with the TB, because every HIV patient screened for TB and as we go down to validate, we look at that data. How many HIV patients were actually screened and there is a form. But we don’t have that form for depression.

R who pays for that form?

MoH It’s a ministry of health form

R So you are saying we should have screening… that form which shows the steps health worker should go through before he screens for depression. Is that different from the other two forms- PHQ-2, and PHQ-9?

R No she is saying there is a standard form now that the ministry recommends. A standard form for TB; TB screening not depression. So we may have to develop one for depression.

PIWhat about.. so you are saying that what about doing one for depression. And all we have to do is to put the nine questions of the PHQ-9. And now that the guidelines are saying this we can look into this and say we recommend that at least once in every six months someone should be assessed.

R Professor from experience – I think it should be at each contact - every visit.

PI So for depression we can put in only two questions so that we don’t overload the health workers.

R Professor for the start with depression, when you assess them for the first time, those with the score -you want to take them to the next stage. You have to follow them at the next stage. But if somebody has that problem because you have been talking about it all the time. It is that person to tell you… “counselor , am I not going to see the doctor” because last time you took that person somewhere and the problem was handled. At a certain level. If that person has that particular problem should demand – “counselor, will I see doctor for this case” because you assessed it last time – she scored at the triage desk…moved to the next stage and she scored that way and she is now on treatment. Being that she is getting some relief now- she will be requesting – “will I see doctor for this case”. now for PHQ, we were also using PHQ but they had…

PI Which one were you using?

R PHQ-2 at the triaging desk. That is what we used for screening, then in the clinical room there was PHQ-9. It had like 15 questions.

PI PHQ-9 should have nine questions. There is also PHQ-15, which talks about somatic problems

R We were asking two questions but we would twist it to get what you want *“owulira nga oli mwenyamivu? owulira nga…nowulira nga oyagala kwetta? Nowulira ngo omutima gukwenyisse?”* with some more questions when you score like four outside, then that one qualifies to see the clinician. One would tell you –I don’t have any problem – I just take my drugs am ok.

R oO.k because like if you saw that tool we have there, one of the things we said is you have to screen for suicide. Because if someone is suicidal- that is a psychiatric emergency. That person actually can no longer be managed there – she has to go and be seen by a psychiatrist. Its ok you can screen for suicide. That straight away takes somebody to stage four. To be attended to by a mental health specialist. That person can die anytime. You are right- I think there maybe PHQ-9 should also screen for those additional problems, but also you raised another issue. You said somebody is aware that they have this problem. So we may have to create demand, we may have to create demand among patients to make them aware that depression exists. so that ..even yourself you may feel that all the time am sad – you can take yourself to the doctor. Am you say I have an illness called depression? We also need to create demand among what…especially during health education – that’s what we are doing for viral load and now people are demanding – “doctor how is my viral load?”

F1 And how do you create the demand?.. how do you create the demand?

R You create awareness among persons living with HIV.

R It will become a topic among the topics we handle during HIV education talks. You sensitize them.

F1 So you said among talks

R Yes in HIV education talks we give health talks in the morning. We make it a topic, because as you are going they will ask today what are you handling –am in family planning, am in viral load… am on missing appointments… it depends though you go out knowing what topic to handle that day

F1 How will you know that you will have created the demand when you do the topic or the sensitization? What will be .. for example

R They will come to you. Mr Ssebunnya those people after HIV education... it depends on what you have given that day. Because there are some where you say ... I have told you everything but you may not feel free to tell me from here – now am going in the counseling room – you can come and tell me – if you don’t want others to hear you. They will come and tell you that counselor for sure me am A…B … C.

F1 So you expect the number of self-referrals to go up! So, indicators have to be mentioned during health education – people will do self-screening and say maybe I have depression.

R Hah, but with the number of self-referrals going up, we are giving the health workers more work. How are they going to cope with it?

R That’s why I asked you – shall we train some clinical officer strictly for depression because they will say “ahaaa this one is for musawo who because they went and trained” – a person will come with the problem –we have identified- no body to work on her after our step. At our stage the next stage there is no one because somebody who was trained to give the service is not there.

PI That’s why in this model, we are suggesting management- we were suggesting depression can be treated by two ways. You can have medications or drugs or you can have psychotherapy. So we are saying in that model that maybe can’t we train expert patients? Can’t we train members of the village health team to provide this psychotherapy in the health activity program. Then we are saying we want to give the patient options. Some patients don’t have …..Because psychotherapy you have to go through eight sessions and what. Somebody may not have time and may say you give me –so we are giving the person options… either we give you a pill-antidepressant. You just get your medication and swallow or you go for psychotherapy- you talk. You go to a trained either expert patient or member of the village health team. To help you analyze your problems, look at the solutions, look at the issues that are bringing the depression. Because we are saying the doctors may be overwhelmed, the clinicians may be overwhelmed. I see you shake your head –can you please share your experience.

R No no. Apart from being overwhelmed, am looking at the situation where there is a mother who needs my help. So, I have to choose, should I see this one or that one...I don’t know how I could put it.

R No we are saying medicines- clinicians

R I don’t know about psychotherapy also

PI O.k under the MANAS intervention, there are therapies which a man like this does [points at a psychologist], those are very complex, but we are saying can’t we …in MANAS they did it – they cut down to … they use behavior activation, simplified –given by lay persons. Actually many trials have been done –where lay person have done it and it has shown to work. Those are some of the things that we are suggesting.

F1 Some are actually just social approaches that have for a long time been done by people without knowing. I think we are moving. Have we talked about the health workers competence? Has it featured somewhere?

R Yes, we have talked about knowledge and skills

R Yah, they are broad. And you find a nurse who is more competent than this [not clear].

R The psychiatric nurse is more skilled.

R Yeah even a general nurse maybe more competent, because competence is about routine……have we talked about the time?

F1 So how are we going to do this? …how are we going to improve the skills?

R But it requires training meaning that they’re easily transferable to the ..

P How about the VHTs we have talked about them and they have skills

F2 Somebody is saying that the VHTs

F1 Yah. We have put them here.

F2 Materials, tools … those are the screening guidelines. we have called them guidelines. There is a question somebody raised- saying that what if they spill the beans. Do they fear that expert patients may tell the secrets in the village? So that means we have to be prepared- they have to be competent.

R Expert clients will always keep the secrets for people living with HIV

F1 Do we have expert clients in the system, are we using them?

R Yes they are already there.

F1 If we have them and we are already using them they should be – because I was about to ask- at your health centre three and fours, on an HIV clinic. How many health workers attend to patients? Because we have some facilities where a nurse is attending to like 70 patients on a particular day; something we need to look at. Otherwise we are going to spend a lot of time brainstorming and generate good ideas but when it comes to implementation…

R You find a nurse who can’t even ask the two questions… she is overloaded.

R They always complain of workload

R It’s about 50 to 70 clients

F1 And how about on the special clinic day? How many patients will you have?

R There is a day we get like 200 plus ….100….

F1 And how many health workers are attending to them?

R Like two and a nurse

F1 Eeh madam DHO! And this is at a Health centre IV? which works as the general hospital

R It is already being upgraded to a hospital

PI And who does triage here?

R The understaffing is serious. The triage is supposed to be done by a nurse; but at our place, it is us [expert clients] who do it. That is the reality

R Well, true. Its expert clients at the triaging desk. They support us. Otherwise we wouldn’t manage.

R These people are playing a big role. The question I was asking her this morning and even Mr Tarsis is here …how ….these people you are making them work….they don’t dig… they have come to work.

MoH But what has happened is that some of them have been rated [remunerated by] by IPs – implementing partners – the IP money is government money my dear. So the PEPFAR support we asked the implementing partners to give them some allowance. Other are paid monthly, but of course it’s little. We have expert clients, like in testing we have RCT volunteers who are there to support the HIV. We have …others are doing linkages we have others we call peer mothers. Like in PMTCT. But we also you can’t remunerate all of them. So they usually select a few who will come to the facility more often compared to others. So they get some stipend……. something little it could be 100,000/= a month depending on …

R Its 96,000/

R it’s little?

R Of course it’s little …it’s better than nothing

PI And fortunately for us during our project, we have put in something for the four health units- two persons. But we shall also give something to the health workers.

R For the case of implementing partners, we are limiting 1 per 150 clients for the health facility –because you are saying the number of clients at the health facility have been overwhelming. So the others are being turned to the civil society organizations and are going to be supported to link and follow up the –people who are lost in follow up. So the civil society organizations need to be ….[not clear]

R So they are going to be tasked with tracing for follow up and then they are going to use some of these programs

R If we roll this out and the program is successful in Mpigi –but the issues of sustainability we are almost sure that the facilities are understaffed and they have to rely on the expert client. How does it feel at that level?

MoH Every district in Uganda has a PEPFAR partner – these projects run for 5 years… 5 years …5 years. At least until when PEPFAR says no more money to Uganda.. this is a function we think they can continue supporting. Of course priorities change but for now it is now in the structure but can only be supported by an implementing partner because you can see with our issues – there is even a ban on recruitment of the technical staff. So this one is a function that only a partner can support. So like now we have new projects that are running for the next five years. So for the next five years we are sure unless Trump says otherwise but we are sure these people will be there.

F1 So in every district you must go and look for the partner

R Every district, yes has a partner. O.k every region has. So they support all districts except that some districts are more supported than others. They are compared on high burden.

R So which means that it is important that this depression awareness is made to the partners so that it can allow the community,, the expert patient to

R Both the implementing partners and the development partners- for the development partners we have like two major one USAID and CDC. So all the partners we have are getting there fund either through CDC or through USAID. Some of the big decisions are made at the embassy. Implementing partners are now the projects…MJAP, RAKAI, RIGHTE, RIGHT EC, ASSIST, EGPAF

R what are the implementing partners?

P The implementing partners… these are now the local mechanisms for example we have EGPAF in south-west.

R What is that

P Eizabeth Glaser (EGPAF), MJAP where we are seated … Yeah, We have intra-health, intra-health is covering the whole of East including Karamoja. We have Baylor Uganda covering the Rwenzori region, all those districts.

F1 Covering which district?

R Rwenzori, yes and also there others, Rakai here, Rakai health. Actually MJAP is a sub grant of Rakai. So Rakai has all these central. We have MildMay. We have IDI.

R Even here?

P IDI is doing the whole of Kampala. Those key partners…. along the way…. we shall have to engage them.

R So that they allow their community workers to be equipped with questions.

P In this particular region where we are piloting

R of Rakai

P Yes. So going forward, i think we need to insist.

P NCDC…..ok…. MJAP, their development parts

R Ok....these are…we have the development partners.. we need to understand the community structure

P The community staffs, health facilities, staffs

R Thats why for us in depression….that’s why we are bringing in experts patient. We were worrried that theses people not going to participate but then this is assuring us that through theses mechanisms, its atleast five years funding from these from these people.

R Expert clients helping out at the…

R They are very much willing

R For them but they are there but like i said we are no longer able to support them in terms of funding. But they are there but also remember that the community health extension workers are supposed to be coming on board and those ones will be having a salary.

R Yes

P Community health extension workers –CHEWS.

P VHTs do not get salary.

R Will these ones have salary?

P Yeah those ones will have salary.

R How many per….how many per…what are the numbers like?

P so they are going to be like.

R VHTs

P They will be like- for them they are replacing VHTs and Health centre 2 staff. That is the plan. Some health centre 2s will become health centre 3s and health centre 2s will be phased out. That’s the plan that ministry has. For them they will have a salary. But that sounds like a long term in terms of the challenges we currently have. Yeah so the long term plan is to have those

P So their function will be like Health centre twos

R Yes their function will be like Health Centre IIs but also because they leave in the community.

R We are looking for money to train them- they’re supposed to be trained for a year. There will be a course….kind of like nursing assistants that we used to have.

R Locally…..Local people from that community

F1 Is there a Curriculum?

MoH Yah the Ministry of Health has already done alot. What is remaining are just funds

R We hope there is mental health in their curriclum.

F1 How can we bring them. He has raised that need that there are gaps. So how are we going to address that?

R By policy

R Ok

R Yes- this is an arrangement for training

R I think at these health centres, there is no specific vacancy assigned to the HIV

R There are some that are there permanent because remember there are gaps every other time- because even transfers -we keep telling them- if you are transferring a clinical officer who has been an HIV specialist of the clinic, bring another one from another facility who is also good at HIV . We really have like teams of nurses and clinicians, who have skills and knowledge. If you look at hospital- the hospital is so huge. Today you can be in Paediatric ward, tomorrow you in HIV. Even in hospital rotations are carefully done

R I have seen that in some hospital- always doing transfers and the people involved are psychiatric nurses

R For me am looking at a….as part of this package- there should be a module to take care of these different cadres- we can have one for the clinicians and then a simple one for the lay people. Lay people am talking about…..

R The clinicians and…….

R Yeah clinicians and nurses

R Do the nurses prescribe?

MoH They do! Our ART programme is nurse led. Actually Uganda was one of those first countries to demonstrate that nurses can manage ART. Uganda trained all these other countries Nigeria where............where….. So we have a nurse led ART program. So our nurse, work as nurses/clinicians we have a module for those and thenone for the lay people. These people were talking about expert clients. I dont know what we shall call it. Community health workers.......... Yes…..So we can achieve that at the end of this phase. Because we are developing a package, so the package should be comprehensive. Should have that…a set of tools, and things …the what to do and how to do it. It wont of course be a long training. Even three days can be enough for a clinician……Yeah……Maybe another three for community health workers. At most like for four days. Yeah- just a small package, training package. Then probably going forward that can be integrated but to start with, it can be intergrated in the comprehensive HIV care training. But to start with we need first of all to demonstrate that this is it that we need to be able to manage depression. Then from there, how do we then make it part of the existing capacity building efforts such as the comprehensive HIV training-like we have the thirteen day training for naive health workers. The ones we call ART-naïve, those who have never had any training especially those straight from school.So like in that thirteen day training, eventually, we should have this module well intergrated. So that when somebody goes through it, they may have it all.We have a thirteen day course for the ART Naïve.

R These are the naïve population-because we are competing for 13

R Those are naïve. The in-service, the health workers that are already working in the ARTclinic, those ones-we are talking about 3-4 days. The 13 day, we were only thinking of now integrating…having…getting that module…. This 3-4 day and putting it there. The only challeng with it is that partners nolonger have that money for many days. The shorter the training, the better. And if you say it is facility based, the better.

R Facility based is ok but you can't work if your clinics are heavy

R But it’s quite demanding. If its done at the facility –many Health workers get involved

R If you have it at the facility it’s beneficial but the manager is crying

R And they do't want to attend because there is no allowance

MoH Eeeh! Thats the disadvantage. The partners are dodging allowances

P The health workers also feel demotivated

R Yeah - It's like for us bringing this training or this workshop here-we had to say if people are coming from Nkozi otherwise if we were to say the health centre is in Mpigi. They would say no ..no

MoH Ultimately, this package should be cost effective as possible otherwise we are now looking at local financing. That’s now the language of partners. Financing, ownership, performance Based Financing. So in the next five years

R They are withdrawing

MoH They’re not withdrawing, but the resources are reducing. The resources are reducing ultimately. We need a model that is cost effective as possible. A model that Uganda alone can sustain.

F1 Exactly. And thats where I still have some a concern. Look at a typical clinic day at a Health Centre III where you have a clinical officer and how irregular they are, the rates of absentism are high. So you have this registered nurse or enrolled nurse. Ok lets say two nursing officers in a unit and here you have 70 or 80 and maybe you have expert clients as she said –they are readily available, how do we see this happening the screening of these patients for depression then handling them, let’s assume they are five or eight-how do you see this happening in real life.

MoH But you see Joshua, the mal-functioning of the systems shouldnt be a basis for planning. Because these expert clients that you see- actually on a daily basis-they are the ones weighing patients, they are doing MWANA, nutritional assessment. So there are alot of things they are doing already. So this is just something small.

F1 Yeah, but when you talk of a mal-functioning of the system, it has to feature in the planning process. Otherwise…

PI Now Taasi, what he is saying: right; As I have told you, I have screened- that person qualifies to the next stage but there is no body to attend to herat the next stage. Eeeh I have screened at the triaging desk and you qualify… Taasi because you have explained- next stage. And you dont have to lineup by the way, the moment I get you, just pick your file and direct to the room and nobody to attend to you, what next.

R Actually now somebody should be able do the PHQ-9. The person at the triage desk first does the two, then now we want to train somebody… another expert patient whom we shall equip with psychotherapy skills and also maybe the PHQ-9, so that she then does that… gets the score and decides..... If this person is sucidal…aha –put on the Ambulance agende Butabika or some other place. Or maybe get somebody and say “this one we can manage, we can give psychotherapy or if the person is comfortable with psychotherapy, we can give antidepressants”. So should I send to the clinic to give antidepressants or if the person is ok with psychotherapy then this expert patient has to sit down and administer psychotherapy.

MoH And actually Joshua we shouldn’t be worried- the moment CLAD starts functioning. What am talking about-CLAD is Community Lead ART Distribution. The stable clients are going to form groups and they will be meeting at the community. In CLAD, they are only supposed to meet with the clinician once in three months. That means if you are stable all through the year you will only see a clinician four times a year. So that’s what I was saying- that simple package of the lay people. The CLAD leader who is also an expert client at the community will be now the one to administer that- because routinely, they will be identifying those with issues - saying that now for you; much as your 3 months are not yet, you need to see a musawo in their group.

F1 So you said the CLAD leader will be doing what?

MoH There are basic things that the CLAD leader will be doing: like documentation because they will be giving out drugs. So they have to document. So thats what I was saying, they should be able to do some triage there. So if we have like our…..PHQ-2, PHQ-2 can now be done there at the community. So eventually we are going to see the facilities decongested and ofcourse donors are very excited about CLAD because it's going to reduce its costs.

R Is there anybody going to collect data-any research organisation which has undertaken to work with you?

P Yes TASO has already piloted. So now we are going to implementation

R It has already piloted it?

MoH Yeah it has aleady been piloted by TASO, IDI. It worked

R CLAD

R Yeah

R That one worked. We are the people forming those groups in the community. So which means the facility will be decongested, so that clinicans only see people they must see

R Yes- Others will just be another category of clients -such as adolescents, -we don’t want adolescents in the community, because of their issues. Even pregnant mothers- If you're an Adolescent, you are a pregnant mother but you are stable, you will still come at the facility, but for you- you will get what we call a fast track refill. First Track you just reach the triage- If you have no issues, you go to pharmacy and go home. Yes -No lining up. So that Jude[clinician] only sees those clients that really have clinical issues. thats what is going to happen.so the Issue of human resource is not going to worry us.

F1 is that happening everywhere in Mpigi

R Yes all partners

PI What is the time frame?. When is it going to happen?

R We already have a TOT this week. Another one is comimg -so as soon as next week, partners are going to start training. they will not start with those facilities. They will identify high volume –high volume facilities and also they will have to do an assessment of their clients, so everytime a client comes- now they do an assessment. Is this one stable or unstable? those who are not stable -they must see a clinician every visit.

R They were making them into clusters of six ..six.

F1 Who is making them into clusters now?

R For us the expert clients, we help them and themselves we train them how they do it. If you are the same parish. for us our role as expert clients is to check your viral load. Dont you miss appoitments? are you ever attending?, whats your viral load? that you qualify, you may even remain home because we said, their six people coming from Kafumu. Kafumu is very far. everyday you use 15,000/- when they are coming and you are six people. Every body will have to spend 15,000, so you make a cluster of six, we assess the six people

F1 So each CLAD is going to have six people?

R Its what they told us, for the start; for Mpigi

R Anywhere between 6 to 10

R For the start, six. We assess that every body is standard, viral load is okay. Though we make it in cluster one will come. for the six we all contribute little money- we give our friend transport and lunch, we save. she comes-we meet somewhere-we get our drugs, next time another one will come. But when you are sick you have to come in-person.

P And these are drugs for three months.

R But you are saying these ones recieve support from an expert client?

R Pardon

R Does anyone go and…

R We are all coming from far but we all sit and agree. How much do you use everyday. How much do use everyday. “I use 9,000/-“. That means the six people will have to contribute the 9,000/-. So we sit and get the nine thousand for you, and we get you lunch. You come and pick for all of us. In the evening, we meet somewhere everyody will take his/her medicine. We give you when we have written the names, tarsis, James, betty, like that. So the next time, me who came last time will not come, i will stay in my garden someone will come in my place.

R So each group…. we are standardizing it. each group will have a leader among them. so that leader is the one who will be doing the basic things among them, trying to see who is depressed, who is not adhering. who is yes...

P The CDDP program. you call it what? its the same as the CDDP program ...

MoH Yah..its the CDDP, but now they are standardizing it. Community led ART Distribution. CDDP was communitty drug distribution point. its the same thing. Of course they came and picked it from here and then they brought it as DSD.

P Yes differentiating, then the muzungu came and picked it.

PI Who initiated it?

MoH It is a brain child of TASO

PI So the differiating is going to fade away.

MoH No for them the differiated is what they have baptised it.

R The bazungu?

MoH Yes. Differentiated service delivery

PI TASO had called it CLAD?

MoH TASO had called it CDDP-community drug distribution points, now the other one has come with DSD. Even HIV testing… DSD, but the outreaches whee started by the AIC. When you look at the HIv testing differentiations, it’s nothing but outreaches, home based, which home based again TASO started here. These are our things but of course now they come in other schemes

P Now we have the guidelines, we have done a curriculum we are doing TOT, we are going to role out .....

F1 May be as we wait for lunch, lets continue because there are other issues that have not yet handled....Madam DHO….. Remember we had asked ourselves- what we expect to see happening –we have mostly dealt with health facility level- have we talked about health management or organization level. What should we expect to see happening at the health management level? Of course these are very nice ideas coming out. And we shall try to simplify because the more complicated we make it the less likely it will be…..we talked of integration. What should be expected at the district health management level? What should we expect to see happening at the district health management organization level as regards integrating

R What you should expect- they are supposed to make and they are supposed to validate. Maybe like how many of the HIV positive have depression. And then validating that and seeing what treatment is given and see our capacity to ….kind of cascade it … whereas you are interested..

F1 At what level is drug procurement?

R Drug procurement…the Health Centre IV order but within a limited budget, the other facilities they receive already made boxes. They’re given a push although once in a while we sit down and say- let us change this to this but within a fixed budget. So it may not make a very big difference. But also at times what we decide to change- doesn’t.

R For example what antidepressants do you have there?

R It’s not enough

P He has because he orders, but the other receives.

R I think most health facilities get the anti-depressants but because they misuse them. What we have seen in other districts…

P But how many tins of amitriptyline?

R But also there is a provision of redistributing the medicine within. Health facility X has a lot of it and the other one is lacking. So if there is that arrangement within the health facilities really

R so how about …I think that’s why we are offering psychotherapy. Because psychotherapy is a good option. Psychotherapy has been shown to work.

R Madam DHO I was asking, don’t you have health facilities having chlorpromazine almost expiring on the shelves. I think most of the ….. am saying what I have seen in other districts – well they may consume – they may complain of the anti-epileptic and antidepressants but they have the antipsychotic medicines expiring. But if the budget is already fixed- why don’t you ask for more of the antidepressants and less or no ..

R Can you change NMS

R Others are saying you can change but within that budget

R Yes but how often?

R It could be that it is tiring because people are missing out the basic

R Because most of the patients who need it may have gone to Butabika. I think this is not far from Butabika. Most of those who need the anti-psychotic may most likely go to the higher level

R Is there functional out-patient psychiatric clinic

P Yes. Run by a psychiatric nurse.

R We have not talked about providing a referral. We have not talked about referal because this model, we are supposed to have….actually integrate all HIV care in the district.

R How many psychiatric nurses do we have in the hospital?

R Buwama has how many?

P One

F1 Next time you are recruiting, it will be nice you recruit more psychiatric nurses because they can work anywhere. But some districts have them even at health centres IIIs, yes - because they realised that this psychiatric nurses have even an added advantage.

R She can work in a theatre. But can also work in mental health.

R Then in mental health. They can do very well in an HIV clinic. Actually they do better in HIV clinics

R Counseling they do it very well, Yes

P They are all round

R So these people are supposed to. So actually we are going to…. How can they provide referal support depression management in HIV. Can they support the other units?

P Under whose budget?

F2 So there units units can only reffer... refer them to you. Do they have aspecialised……. or specialize a day. Do they have a role by the way in HIV MANAGEMENT?

R Yes, for example some of them are very negative though they have been trained. Some of them are really positive

R Positive about what ?

R HIV

F1 You have raised some good point there. You said some psychiatric nurses are negative about HIV. Ok you were still telling us

R Some are very positive personal psychiatric nurses

R Upto now we are seeing very litttle connection between psychiatric services and HIV, How ca we improve between HIV services?

P Ok, We are trying to improve mental health.

R You said that some of them are not negative

R Some of them are not.

PI They too have the negatives- We have to address those negatives. Actually you may find they are not very confident in HIV management. So they may need a module as well. A training.

P Yah, the integrated… a training that makes HIV and mental health talk.

R Before you start seeing referrals to, you need a training that targets them.During training we need to give them the right support

R But the good thing as doctor has said for Mpigi health center, one is now the in-charge but also inBuwama that one who is there – also is in HIV clinic. I don’t know whether he is the in-charge but he is there atleast.

R But I think training goes with supervision

P Yeah Attitude

R The attitude bit can be addressed through supervision

R Now who supervises? Because we cannot now say the centre… we cannot say Butabika- we don’t have resources for that. We want supervision to be preferably within the district. How is Nkozi? Does Nkozi have a mental health department?.

R They have I think it’s done Monday -weekly

R And they have a psychiatric nurse probably?

R Yes they have

R Is it an outreach?

R No at the facility

R You have to pay for the services

R Pardon

R It’s a mission hospital; a PNFP.

R Yeah

R Its PNFP government supports them. They should be subsidized. It's like Mengo and Rubaga

R Mengo you pay –millions!

R But the ART and related are subsidized

P Aha subsidized- the word of the three letters remains. They pay. How much? But they pay.

R Though it’s hard, we need to think about supervision. Either it’s a Peer model

R Maybe we will need someone who is a focal person on that the HIV focal person, what about the mental health focal person in the district. There should be someone who report to the DHO or somebody we shall ask- how many psychiatric nurses are there. Bla bla bla. That person should the link preferably a psychiatric clinical officer or a very active psychiatric nurse.

R You have also reminded me, the issue of coordinating the depression management in the health care.

R But should be knowledgeable

R Yeah, that's of course. Ok. If he is not psychiatric. You divide the powers too much- You put a nurse to head depression. And yet we are talking of integration.

F1 Aah... Madam DHO, the issue of having a focal person in the district. I hinted on it last time even now we are talking about it. I think maybe you get the psychiatric nurses you have in the district, appoint one depending on the… Forexample we have worked in Kamuli where there was one PCO and automatically he qualified to become the mental health focal person. So when we sat with the DHO and the psychiatric nurses. It was the psychiatric nurse who was more active than him. And I had taught this PCO. He was appointed the mental health focal person but he is hopeless- so somebody else doses the work. A psychiatric nurse does the work.

R Although now we are talking about HIV care.

R Yah. HIV care but depression… now is mental health

R No but you can't split too much.

R When it comes to referal, how about referral follow up and supervision. Some supervision and follow up to link with the HIV managers

R It has financial implications

R Yes because this person has to move from his or her unit.

R The only person who has the capacity- the only person who has the money to supervise [referring to the DHO]. So you better equip the whole…

R Not focal person. But supervise the ART services, you also look at depression.

R True! That's why my capacity has to be built. The capacity of the DHT, so that we also look at the need to separate anxiety from people.

F1 HIV, TB focal person, who, but… Madam DHO, this would be some form of delegation. Have some psychiatric nurses you are saying

R But you see we want to improve her capacity before she can supervise the focal person. We donot want to create more and more focal person for TB and HIV

R My worry is we may want to have management of depression and end up with mismanagement, some patients are given carbamazepine instead of chloropromazine because the person doing it is not.

R Then improve the capacity of DHT

P But we can also co-opt in ……at times if he is somehow supported

R Expect the district to do the same

R They expect the region to do the same.

R Which regional referral takes Mpigi

P Naguru

R But i remember Rakai-Masaka it was through HIV/AIDS program without splitting

R How do you supervise them

R TASO

R The nurses were doing completely

R Those were psychiatric nurses hired by our project

F1 I dont know whether we have exhausted whatever we are meant to cover

R What can be done, CAO through DHT

R CAO's office possibly to be instilled to monitor issues of health

F1 Building their capacity

R Yeah building their capacity, sensitizing members

R You also look out for neutral issues

R As they sit in their monthly or quarterly meetings they can possibly discuss matters related to depression

P Baayogedde ku domestic violence. Nawulidde agyogelako -Bigwaayo ewuwo nnyo

R - With that we talked about the personnel. Though scattered- there could be some other that we

R - Do we see the role of the bio-statistican?

P Definitely – bio-statistician

F1 - Do we see any of the district bio-statisticians in this?

R at the end of the day he has

R - Is he attending any of our next meetings?

P - We have oneon the district, Not in this meeting

R- Is he attending our next meeting?

R- Since we have saved our, then we an have him attend our next meetings

R- We were worried about the power relations -So we want people to speak- Actually even her she won't e around but we want the practitioners to bring out issues

P they will speak

R wont they fear you

F1 - Yes. When the proposal was done,- We assume there will be power relations. So that’s why they wanted you the managers to be here, the other people……..but for her she can be anywhere

R Agreed professor

R -and the good thing when it comes to working with those four health units -we are going to go and have meetings there. So we shall meet them again. So don't worry, when we come to the health units. When the project comes to that level. Down there we shall introduce the projects……someone ooh We have to engender, this thing has a gender component and i will be asking about another woman to talk about- we must engender our program

R Gender mainstreaming

R We always look at the gender component

R Planning but of course planning includes budgeting, implementation

P Issues of adding staffs like environmental health staff here in the community, when it comes to issues of following up VHT's. I think 8 have been following up the VHTs. They are also attached to the facility part of the training.

R the environmental health?

P The environmental health workers-

R - It is these detectors that are attached to the training

R - It comes to issues of the training, leave them out. Community health workers, those are the health assistants and health inspectors as we may take them. They re attached at the fascility PHT. All these programs

R - Some other comment?

P - About the training, during the training, question or even other

R - Guidelines, question guidelines

R - Bu those are training tools

R - If you are going away with some things unmasked, please unmask

R - I think let's clap for ourselves

R - Some of the assumptions that imply forexample

R - The most important thing we shall do is work on everything
