# Supplementary material for: Using Theory of Change to inform the design of the HIV+D intervention for integrating the management of depression in routine HIV care in Uganda
Source: PLoS One. 2021 Nov 30;16(11):e0259425. doi: 10.1371/journal.pone.0259425 (PMC8631669; doi:10.1371/journal.pone.0259425)
Supplement: S2 File — (DOCX) [file pone.0259425.s003.docx]

**Preliminary Theory of Change Workshop (ToC) 2 in Mpigi**

**Attendees:**

MRC staff

- Joshua Ssebunnya (F1) - Prof. Eugene Kinyanda
- Richard Mpango - Dr. James Mugisha (F2)
- Dr. Maggie Nanpijja - Kyomuhangi Ruth

Participants:

- Health workers
- VHT members
- Expert clients

Introduction.

F1 As said earlier, this is a research workshop. We want to get feedback on the need and acceptance of the proposed interventions. We also need to get feedback on the feasibility of the intervention and have insight on the anticipated problems and possible solutions in the course of implementation of the intervention. We want to share ideas with stakeholders and all of you are stakeholders, there are health workers at different levels, patients and lay people. So we want to work hand in hand to see that we are not imposing the project or interventions on you. The intervention we are talking about is one that will eventually be adopted by the Ministry of Health; but before it does, MRC is first testing it out here in Mpigi.

The intervention is tried in Mpigi then later will spread to other districts, like 3-4. Then later the Ministry will take over the intervention and scale up fully. We want to know; is there need? Is it acceptable? How will it work? What hindrances may be there? Because it’s good that whenever you’re preparing any program it’s better to first identify the hindrances you might find on the way. If the problem comes how you will overcome it, you must have prepared, thought about them and you prepare for them, so when you come to implementation it’s easy for you.

As we all know HIV patients,…..the project is HIV+D because as we know patients with HIV sometimes get depressed due to so many issues. So the need to treat depression is there among those people because it’s there. We did not just come and say people with such illnesses tend to have depression, no; this was first researched about. Many people conducted research and found out that people with HIV at times have depression as well and it usually goes untreated. Now on our agenda we had the objectives, the intentions of this workshop we have understood them. Why we are seated here as some of the stakeholders is to share ideas on something we are cooking….we want your contribution on how we should prepare it, what should be added; instead of us coming from there and call you that we have brought a program; and tell you that you should do this and this. We may have brought something but they may not be the best. So that’s why we want to involve you in the preparation of what we are preparing.

That consent form which my colleague has taken you through has provided you enough information about the HIV+D project. We had the launch here still this month. At that launch we talked so much about HIV+D, so most of you know it and we’ve talked about it again. The program HIV+D, is a program like any other program you would name….we are calling it HIV+D. There is HIV, which we all know. Then the D stands for depression. As already indicated, the aim of this program is to incorporate treatment of depression in the management of patients with HIV. Something important to note is about depression. Do we know the symptoms? Is it one of the illnesses we often talk about? This sister has talked about it. Is it an illness the community knows? Have you treated anyone saying am sick these days, suffering from depression?

P- He/she may not fall sick but can be there and say “I am not well I have too much thoughts, I think I am sick of depression.”

F1- Okay sometimes we used to use “sick of depression” it’s the term we used because it’s what people know. You cannot talk about depression in Luganda and don’t use the word thoughts. Yet it’s hard to find someone who says “I am sick,” what are you suffering from? “Thoughts” they never say that. When do you get those that say I am sick of thoughts?

P- They say I have thoughts.

F1- But us don’t we have thoughts?

P- We have

F1- We all have thoughts….this is something I want us to understand. Most of you treat patients with HIV and TB. It means when the patient comes you check for symptoms of TB and you treat both together. This thing we add for depression is not easy like people would think because if I say “I have thoughts” we all have thoughts… even the doctor you’re telling has thoughts. But for him, its not depression. We must understand clearly that people don’t even talk about those thoughts as the reason that brings them. The truth…. the expert clients may tell us; when its first time for one to know that he/she is HIV positive, the heart breaks. One becomes sorrowful and could say “I have stress, they told me this or that”. So basing on the stage at which you’re and words coming from people, some will not even talk. That is why to find that someone with depression will not be easy. It is not like how some people may think. So, for this HIV+D….Professor Kinyanda who is our boss is not here right now, but has done research in this field for some time. He is a specialist in mental health but in one of the studies he has done back in the days found that depression is common among People with HIV. He did not just dream of such a project. It is informed by earlier research. So it’s a fact that depression is common among people with HIV, but what is being done currently? What are health workers doing at our facilities to address it? You find that on the HIV clinic day, you find the health worker with like 60 patients.

P- 60 is little. They can even be like 200 or 250 patients

F1- Attended to by how many health workers?

P- Two or one

F1 So when they come, what do you do for them? The truth is we don’t do much in most cases. Now there is what we call common mental disorders. These ones, we all know the word common. There is the depression we’re talking about, anxiety, they’re the most common ones. Of course not only these but there are other mental illnesses. And if we talk of depression with mental illness, we shall not talk of madness…. someone talked of madness, but this person is not mad, and may not need to be referred to specialists or Butabika. So we are talking of disorders we can manage at our level, which don’t require us to refer to Butabika. Common mental disorders are common but are usually not detected. Sometimes it is because the health worker does not have enough experience. Sometimes it is because the signs are not obvious and so they don’t see them and the patient goes. So, the problem persists and causes more problems. For example someone depressed may even stop medication and feels like hating him/herself or committing suicide. At times some HIV patients kill themselves, and in so many ways if depression is not seen and the needful is not done. Sometimes even if one is taking the medicine but is not okay inside… when life is not good, you can’t be okay. And if you have something disturbing your mind, the medicine is not going to work well. So, depression is among those common mental disorders that often go undetected and untreated.

As we have said in the introduction, the MANAS project in India did similar work though it was not specifically for people with HIV but still it was on mental illness generally. MANAS is a term in one of their local languages, but it means ‘humanity’. The project was trying to address common mental disorders in PHC so that the primary health care workers could and treat them, because if they are not treated, they persist for long and the clinical outcomes become poor. Some of you can testify there are patients you treat and don’t heal; we have worked in number of districts and the medicine you mostly give is coartem and paracetamol. Symptoms persist, patients come back all the time complaining of headache but when the underlying cause is something different. You keep treating symptoms but not treating the problem. So, we are going to replicate what MANAS project in India did. But we are not necessarily implementing MANAS program in Uganda. That was for India.

In implementing this program, our focus will be on depression and not all the common mental disorders. Now in this workshop, we are involving you as stakeholders in refining the interventions to undertake. We didn’t want to come here with predetermined things to do.

Now we are going to use what we call Theory of Change in adapting MANAS to modify interventions for the HIV+D program...

What does Theory do change mean? This is different from the usual way of doing things. Traditionally we do research and then identify/plan interventions to undertake based on research findings. We would come and say “in view of our research findings, we are going to do this and this and this as interventions”. With Theory of change, we do thing the other way round. You don’t sit there and decide what you’re going to do. Rather, you sit with those you’re going involve in the work and to determine what you’re going to do, but you move from the other side going backwards. You say “what do we want to achieve? Or “what change do we want to see?” for example now we want to see that people with HIV and depression have their depression detected and treated. So we start from there, identify the change we want to see and move backwards identifying the preconditions, I mean things that have to be done or have to be in place first for us to get where we want to get. We say for this to be achieved, this and this and this have to be achieved first. But for these to be achieved, this and this has to be done; but for this and this to be done, this must also happen, and this and that. This and that have to be in place. We also need to identify things that will enable us know that we are on course, call them indicators.

So, we are going to start by identifying impact or the change we want to see. Then we understand the change and we work backwards to identify the inputs because there is going to be a lot; the activities that will be done. We can also identify the anticipated problems along the way. So, we are not sitting in office and plan so as to come here with pre-predetermined ideas. Are we together? This way, we avoid implementing mistakes. Some of us have worked on projects where we had pre-conceived interventions, which did not make a big impact. For example you do research, find knowledge gap as an issue and then decide to train health workers. Who told you that they want to learn anyway? Or how do you know that the way you’re planning to train them is the best? So, end up planning based on assumptions. This helps us not to implement or avoid mistakes like we have told you we’ve had intervention and didn’t work. You go to health center, you teach them, give them some materials, and when you go back the next day, you find when you they have thrown away the materials or used them to light the charcoal stove. So it’s like you planned wrongly… maybe they did not need that knowledge. So, here we want to avoid implementing mistakes.

We want to integrate the management of depression in HIV care at all levels where they treat HIV patients. But we have to understand that to manage depression, you have to confirm that one has depression because one will not come and tell you that I have depression. After identifying the impact, we then identify the outcomes that will eventually lead us to the impact. Some could be long term, others short term. We have to identify assumptions in all that we are doing and what we are preparing. We have to identify indicators. Indicators are things that are going to show us that we are on track, things we’re doing will help us reach to our target. Then we also identify activities. We can call them interventions; what is expected to be done that this and these are the important things to do. We can as well identify the personnel, resources and other inputs we shall need. Of course later on when handling patients, we have to understand where the depression we are talking about comes from. One would not work on the patients well if he/she does not know what caused the depression. We are then going to come up with a map that links these things…. where we want to get, where we are coming from, things that have to be done, what has to be in place etc. This map can even be used to monitor the program. That is what we call the Theory of Change map. One other important objective of this exercise is having some insight on any anticipated problems or challenges along the way and possible solutions. We have worked in some districts and later regretted why we went to work there; let me hope we shall not regret why we chose Mpigi. You discover later that people you’re working with are wrong people…. in a sense that 90% of the health workers could be misplaced. They were not meant to be health workers. Maybe they had to be in the market selling ginger, others in police. So we hope we won’t regret because of people’s negative attitude.

We are starting with getting the long term outcome….in fact the impact, although this is something we may not achieve during the life of the project. We have talked of integrating the management of depression in HIV program in PHC. The period we shall spend in MPIGI we might not achieve it fully that’s why it may be long term. Other outcomes might be short term. Don not mind about my diagram. It is rough, it is for demonstration. We simply want your ideas now, and we shall draw a better diagram/map later, where they will be reflected.

So, having stated our long term goal, can we now identify the short term outcomes. Some could be intermediate out comes….some could be inputs. Don’t worry, just give your ideas and we shall see where to put them in the map. But we have to identify things that we can see that are there and as you see the arrows are pointing backwards, we work going backwards till we get to the activities. Do we agree on that? Is there anyone with a question before we go ahead?

All- No

F1- is that for this workshop, you’re not passive recipients. We want active participants. I don’t know whether you have understood this method of planning for programs.

All- It is not bad.

F1- It’s not bad in what way?

P- It shows us what you have to do and brings out the importance.

F1- You get to know what you have to do and how you are supposed to do it. Who are supposed to do it? Why this one not the other one? Things will not be by trial and error, these ones will work as you understand what you’re expected to do prepare and come out well. There are four health centers where we are going to work, Mpigi HCIV, Buwama HCIV, Nkozi hospital and Butoro HC III. If the program is very successful your district will be the first… because your DHO is very positive and is involved in the preparations. There DHOs who say “you go and work, the health workers are there” They show no interest except when you tell them time for signing.

So, back to business, what impact do we want to see? What is it that we want see?

P- We want to see that whoever has HIV with depression are treated together.

P- Depression management and HIV care

P- We want to see patients with HIV and depression treated together, not giving you ARVs here and then say “you go to the other lady” yet might not even reach there or even getting problems.

P- Another idea, we want to see that if a person is told that he has HIV, that should not bring him/her depression, thinking about this that, seeing him/herself worthless, etc. Or the handling of HIV patients does not cause someone in that situation to be depressed.

F1- Am seeing an element of prevention

F2- He’s saying someone with HIV does not make them depressed having seen people with HIV living happily without depression.

F1- How do I phrase that answer?

P- Telling someone that you have HIV does not really cause you depression

F1- If I understood well; you want to see that people who have got HIV, even when they have been told that they have HIV doesn’t reach a point of depression. Okay we want to see no incidence of depression in HIV cases.

P- I was suggesting that people who have HIV and don’t have anything paining them, should be given medicine for more months so that they don’t always be looking for transport to come back to facilities.

F1- It’s a good suggestion but is it an impact, where does it fit in depression? Though it’s a good thought we shall get somewhere to put it. That people with HIV are given medicine for long period so that they don’t be coming all the time to facility. Isn’t that what you’ve said?

P- Yes

F1- You have said those with HIV but are well, there what?

P- When they don’t have any physical pain.

F2- That is more of quality of life

F1- That comes here in outcome just that the way he has brought it surely it had an activity, giving out medicine for six months. Okay it’s more of an activity but as we said we are just capturing everything we shall sort them. My colleague has said that what you have told us we can find a way of bringing it here but looking at quality of life for those people with HIV that they are in good health. I don’t know if it’s that. How can we call it?

P- My idea is like this, if someone has been found with HIV and that brings depression mostly at heart because they point at him/her, discriminating because they’re still there. If someone has got HIV may be a child the friends point at him that one is sick. If its education that child is tortured with such thoughts….might even be in school and instead of reading is depressed or worried all the time because everywhere he goes, he/she is the topic. For example can be at school and remember “am like this” so can be depressed all the time.

F1- I have not got that clearly what you have given us… is it a suggestion, activity?

P- I have put a suggestion and an idea.

F1- I think I did not get the activity clearly I beg that you clarify

P- Am saying that if there is educating that child you find that this child gathers together with colleagues that don’t have HIV because most of them are left home and refused to study they say “that one will not go is sick.” Doesn’t have the hope that this child will study and graduate work and survive.

F1- Okay there is stigma that even where they get service sometimes also find discrimination

P- And pointing at them

F1- Should we bring that in outcome? Stigma free services so those are some of the things taking us to impact. I didn’t know that they still do that the doctors also discriminate.

P- Even the common people

P- Thank you so much, I don’t know how impact is changed but the way we can get impact or intervention to see that all those we have started with or we have discovered with depression, we start them on medication until they get well.

F1- Those discovered with what?

P- Depression we start them on medication.

F1- For depression

P- Yeah patients until we see a good progress on treatment.

F1- You’re talking of better clinical outcome.

P- Yeah

F- Improved health, well-being is what he’s talking about.

F1- Okay improved well-being may be broad.

F4- Better recovery

P- For those who have been identified

F1- Improved health outcome or better

P- Better

F1- For those with HIV and depression now before we go any further, let’s first identify these

P- Mr. Ssebunya there is somewhere I haven’t understood our theory of change comes from there other side going this way

F1- Exactly

P- Now it’s like we are just giving general but if we start on impact; what are we targeting in our study because long term if we start outcome then come backward?

F1- That’s where we started but as I told you, if we discover that it doesn’t fit here we put it where it fits but we started from her. We don’t want to lose it if you have brought a point we can’t say “it will come later no; we capture it find where we put until we exhaust we walk backwards. If there is something that has come and doesn’t fit there we find where it’s supposed to be so that we don’t lose it.

P- I have an idea that says that someone can be depressed but when it’s the sickness that has made him/her depressed. We may treat the sickness but we don’t find what the cause, I may be there at work depressed and what makes me depressed the situation at home. The woman what she goes through at home; husband mistreats her, is looking for children’s fees so we should look for cause and address it other than treating.

F1- Now there we are not on impact but you have brought up a good suggestion. We have to identify the risk factors or causes for depression in HIV patients. Isn’t that what you have said?

P- Yeah to include other activities

F1- Actually that takes us to or when we reach here in the middle we shall see how they relate.

P- Another impact may be all health workers dealing with people living with HIV are able to identify and treat all people having HIV and depression.

F1- All health workers treating HIV patients

P- Yes or handling HIV clients

F1- Handling HIV clients

P- Identify and treat.

F1- Are able to identify and manage depression that is an outcome that would eventually lead us to impact. Impacts remember we called it long term vision or it may go beyond depression. There is one who brought something about clinical outcome depression patients managed, incidence with no prevention, cognitive, living better quality of life and we could even go beyond the depression along. What would we target when we talk about managing depression and HIV? What would we want to see? What condition would we want to see there? Sister what condition would you want to see?

P- I would think that it would have been good that the person with HIV is treated well to reach the extent of not having depression.

F1- You would want to see that every person with HIV is treated and handled well so that he doesn’t get thoughts. Should we think that some are not treated well that’s why they get depression?

P- No treating is in so many ways but I am talking about treating of depression that if that person is treated well in that condition and is not getting those thoughts again. Even though he has got the other treatment can be there with anything disturbing the process of this treatment.

F1- I want to ask you some question for elaboration. You say that a person with HIV should be treated well… the depression. How shall we know that he/she has the thoughts?

P- Now the process of knowing that he/she has, we first talk to him.

Now there you have brought in another aspect…

P- Yes

F1- Now who should they talk to and who should be left out?

P- Talking to every patient that has HIV, you have to first talk to him/her.

F1- One of the things you’re talking of is screening for depression in all HIV patients.

R- There is no way you can understand if you have not talked to him first.

F1- What you have talked about has so many ideas we had to first break it. So you would want to see that every patient is checked or they talk to him/her if is depressed.

P- Now after knowing that he/she has depression, they refer to the clinician to get treatment. I am hoping that even though you have discovered him that moment and sent him there to the clinician that can treat him when the medicine is there, it cannot cure that very day and they give him/her time and comes back until he gets better. However to ensure that they are over you have to go ahead seeing him/her.

F1- Help me there might be a point I have missed

P- She mentioned screening, managing and also close follow up until it’s clear; screening until you achieve total treatment of depression.

F1- Close follow-up

F2- She talked about referral and screening, should we also take assumptions?

F1- Assumptions

F2- One of the assumptions in what she has said is that referral system is working effectively.

F1- Screening, close follow-up if we put referral where do we refer? My colleague has talked about it assumption I will use different shapes. We assume that referral system is effective because you may be wanting refer but when the system is poor. You don’t even know who to refer to, whether he/she is there or not. So assumption this will come there in the middle but you were told you we are generating something we shall put in the map the way it’s supposed to be then we display it well.

P- Total clearance of depression patients

F1- Total clearance of depression… here I said improved health outcomesfor those with depression.

P- Not like getting rid of depression

F1- Getting rid of depression

P- When one doesn’t get sick anymore.

F1- Definitely improved health outcome, okay eradication of depression not seeing any depression.

F3- Close follow-up until you’re sure of full recovery.

F1- Okay close follow-up we can have it… full recovery. Sister what would you want to see?

P- Me what I would want to see would be that all people with HIV are taught about depression, what causes depression? What would bring it so that they know where can to run to and where to start from.

F1- Sensitizing about depression, possible causes, resources etc. and that is an activity it’s a good point.

P- I would also want change. There are some of our people who know what they are but they don’t mind to go to hospital to get treatment that is near him/her. Such a person stays there or sending another person to bring like septrin and swallows and stays but knows what is dealing with. Reaches appoint of coming to hospital when is ill can’t help him/her self.

F1- When is not on medication for HIV

P- Yes not on medication

F1- Now if depression gets him, how will we reach such a person and how are going to help him//her?

P- Now to help such a person it needs to get up or get someone who is an expert in that condition and you make him/her love this things so that can come out.

P- Reaching out with support

F1- But these are people with HIV who are not even on treatment at the health facilities, the ones he is talking about.

F2- I think he is talking about using VHT experts reaching out to them that they know because there in the community.

F1- I would assume the expert clients… do you still have people that don’t go hospital to get medication and there still in denial?

All- They’re there

P- They’re many…..

F1- The truth is those ones need them more so how can we help them to see that they benefit from this program.

P- How about if we phrase it like empowering referral systems in place because the experts and VHTs are there? He/she may reach that person and fail to convince him/her well but put an idea in him/her that if I take you to this doctor will help you; that is referral.

F1- So you’re talking of referral and empowering referral system

P- Yes, how is that?

F1- Of course once you have an effective referral system, it has be working.

P- I would want to see… there are two situations that are very critical as regards depression; the way the person is handled before being tested (pre-testing) and what happens after the testing (post-test). Those people who work on that should be empowered enough to see the situation of that person they are going to explain to that they have got HIV, it can cause or prevent depression of that person they’re explaining to.

F1- Before you add anything, that is a good idea. So, he is talking about capacity building. Who are supposed to do to pre-test and post-test counseling?

All- counselors

F1- But these people to prevent someone from getting depression….the way they handle the person can cause or prevent that depression.

P- Now another thing the nurses should be seeing patients and handling them as they are. Sometimes they can ask questions that may make the person feel more sick….and makes the person depressed.

F1- The way you handle the person?

P- The way you handle him/her and what you tell him/her, what you talk about, you don’t want to tell him the truth, he just hears about it somewhere that is this and this. That situation would help if the nurses handle that person as they come. The situation and know that this one is in this class so that reaches somewhere and comes out happy.

P- What I am asking is him to elaborate what kind of class because we can look at class where one has the ability to know. From what he has said, we can say that if I come and you see me… maybe I am smelling alcohol, don’t remind me of it immediately. Handle me well, explain to me the possible problem I may find in drinking alcohol yet I am on medication. I have not understood what class he was explaining.

P- Now a patient has come to your table when he starts speaking you can know it’s just that I have talked about alcohol. Let’s assume maybe patient has come and doesn’t take alcohol but has some days when he missed the appointment and yet is very bad. What if doctor says “are you mad, do you care for your life? You don’t take well the medicine you get this and that. You are quarreling showing anger but it’s him suffering more than you but you show that for you care more than he does. So that way you handle him/her… know the situation he/she is in and you change him/her from that situation but when you’ve not blamed.

F1- Okay impact could be there as an outcome then the assumption is professionalism.

P- My idea is what I would think about is that…….and another thing I would think that these doctors who are helping to have positive attitude because we have had so many programs but people have negative attitude when working on them.

P- What I would want as me is to see all people because when you look at depression management it may be just talking and one is well, then there is that where it may require simple medication. What I would think is that in the situation where we would need medication that person ….. says we’re going to give you something and leaves the facility convinced.

F1- If that person needs tablets let them be available, if he/she needs counseling let it be available. So we want all services practically available. There we are on outcome; services and resources in depression management available at the facilities. My colleague is asking if it’s an assumption but its true some of these appear as outcomes because services and resources appear as outcomes but it could also be an assumption between activity and outcome that’s why we get short term intermediate.

P- I would have wanted to see that whoever comes in is screened without questions and we discover whether one could be depressed or not. One with depression goes through proper channels and is taken to the expert who can handle in any way. After handling that person what my brother has said he/she gets medicine and is followed-up to see his/her progress until. These ones that don’t have we also tell them about depression and how they can live without getting it. I don’t know whether you can pick something.

F1- I have already picked because screening is for all.

P- Where at the facility or community?

F1- No, who can do the screening in the community?

P- VHT

F1- The HIV patients if they come to the clinic it’s where they get treatment so do we want to say that VHTs should go and screen in the community.

P- VHTs are in the community

F1- Some don’t even come to the clinic medicine finds them in the community.

P- They’re there HIV patients…..so it’s like you’re proposing screening in the community.

P- Also at both.

F1- So you want us to talk of screening at the facility and in the community.

P- Okay we can say identifying in the community then screening at the facility or you can empower the VHT and they go do screening when they’re in the community.

F3- Joshua, do you remember the two questions that we had; do you have difficult in sleeping? Do you have appetite or have you lost your appetite? Those two questions can be asked by a VHT at community level and then depending on the answers this patient can be referred to hospital where they have long questions. Depending on the responses said that person triages that it’s clinical.

F1- Okay you’re talking of empowering the VHTs training to be able to screen. Remember even screening is not diagnosis. So, we are not using a diagnostic tool. It is probable depression at that stage because even at health facility you would be screening alone, you can be screening one but its probable depression. In our setting I may be stressed for about three days that’s not depression, I can be with HIV and lose someone I like, in whom I was confiding. I will be extremely sad but not depressed. The work at the facility in fact has to remember but in the training they will be coming.

P- Question but am focusing on screening that we see we’re integrating HIV into PHC now community has different people but those people are not attached to the facility. Won’t it affect in follow-up center or to get the service? Won’t it affect these people because if we say the VHT to screen in community should we say that VHT screens those who have got medication from the facility in that catchment area or that he/she knows?

F1- There expert clients can answer that

P- The VHT’s work in the community may be it has problems because the VHTs in community these people that have HIV don’t trust them at all.

F1- They don’t trust them

P- Yes not at all, there only few that work together with them yet screening would be beneficial at the facility but not community.

P- Meaning it goes back we are going to target clients that come to facility

F1- Yes

P- What he says that we screen even in the community

P- Except when in the community you send expert clients because for them they know and if he has come, they can talk to him well but the side of VHTs from the village, there is war.

F1- What kind of war?

P- Rumor mongering

F1- Yes there are those VHTs that are good and do a good job. VHTs are the chain between the community and health facility. So, if they don’t trust VHTs, it means there is something wrong that has to be put right.

F3- But she gave expert clients as a ready alternative, they are more receptive, are more acceptable than VHTs in this case they’re more prefer than VHTs.

F1- So they believe in experts clients because they resemble them.

P- Yes

F1- What if the VHT is also client?

P- Those ones they believe in them but these one of villages they come and report to you “what did you do to us? I saw the other coming to my home I left her there and walked away?” things like that.

P- I have not understood that issue well but haven’t answered fine the expert clients may be going to the community but screening goes back to you the facilitators. Do they screen whoever they know because they can know all of them? This one is from Buwama, Mpigi now screening does it target clients that get medicine from Buwama or if expert client is attached to health centre IV and we say we screen for depression is he/she going to screen all.

F1- I don’t think the VHT or expert client is going to screen people who are not in that catchment area on that facility because there is no way he/she will help. That screening we should not be so ambitious, let’s not think there is someone who is going to go to the communities and equip VHTs or expert clients because if we go through with Muzeyi on how to screen I don’t know whether he will take that as ajob and start working house to house those that he know and goes to screen them. Do you think its possible?

All- It is not possible

F1- Most likely for screening we shall talk about it at health facility but not in community. Like I have told you even at health facilities, there are some health workers who fail to screen these people right. When you look at depression we are talking about, it is not like malaria. After screening, they need to do clinical assessment to confirm that someone has depression, let’s not think of things that are not applicable.

F2- There is something we talked of in other workshop… I don’t know whether lay people especially in the village know the word depression. Because its a heavy word. So they may screen the people that have problem with the brain and we sort ourselves there. F1-That’s why I thought we should not suggest something which is practical at all……. to empower and strengthening the referral system. Referral system starting from expert clients, if… to refer you at hospital does it need to be screened first because for the hospital we do usually we first screen. You may have told this person “why don’t you go to hospital and they see or you tell his/her doctor because the VHT responsible by the time he/she goes, some of them you find them in clinics. So if we say we put screening in the community, hah! I don’t even know who is going to supervise it. We should not be so ambitious.

P- People who have HIV sometimes those thoughts kill them quickly I was thinking that to completely erase the death of people with HIV……. Someone can die in the period he/she started medicine.

P- Improved survival.

F1- Mortality attributed to…..

F3- Depression

P- Most of the time when something…. So I would think that if we could get focal person at the facility responsible for that thing like we see TB focal person and what have you, how does it look like?

F1- When of the focal person on what

P- Most of the time we report the case to him, is the one responsible to see that the thing moves. Like TB focal person most of the times find him people are like this, how does it look like.

P- Yes its true we say there is one we have identified started medicine they have focal person but they are different there is one responsible for health centre, HIV clinic we have TB.

F1- So what kind of focal person is he talking about.

P- Focal person of depression

F1- There we’re off track because integration, depression management we will not have integrated it in HIV care because if we integrate we don’t need focal person that is responsible for this. When we talk of integrating mental health into PHC, it means we don’t need a psychiatrist or psychiatric nurse at each facility. It means that this enrolled nurse should be trained in symptoms and medicine so that can handle.

P- To handle all.

F1- So we cannot have focal person for depression management and then say we integrated.

P- I had looked at it like we see TB most of the time is screened especially when treating in HIV though they just look at it but it has a focal person who is responsible for it but look at that thing so that it goes forward.

F1- Now, suppose another research study discovers that HIV people are drunkards, shall we get a focal person for managing alcoholism in HIV?

P- So if there is someone who minds a lot about that thing….

P- In fact if that person is there, they will leave it to him

F1- They will leave it to him because the problems we get sometimes with mental patients, if you put a psychiatric nurse, patients come and the other health workers will say “those are your patients”. They always send patients to that nurse. So we have to change attitude and to see that we see the problem and find what to do about it. What caused it? Is it caused by someone’s behavior that they refused? With integration you don’t have to use focal person on everything so that they understand.

F3- Is there focal person for HIV?

All- Yes

P- So if there is and we are integrating depression in HIV, the focal person in HIV will be the person for depression because we handling depression together and therefore in this case the HIV person will manage.

F2- And remember one of our goals is to… if that HIV focal person is involved.

F1- That question has come is it feasible before we go any further? Will it be difficult for focal person?

P- But if we get focal person for depression it does not we have isolated depression and other services. This person can monitor or to make people like monitoring service that’s why we have not said you’re for that so handle everything even TB there can be a person but are working together.

F1- Now like we have TB focal person we have said things that concern him is people of HIV if we start project and every problem should we appoint someone to handle it.

F3- No

F1- Secondly that is not what integration means

P- I would say that people now… where it would help us like we said these are nurses working in ART clinic, they should be trained in depression management. What is it now that is supposed to be treated and what they should do?

F3- Building capacity for all in treating HIV

P- It’s me who has HIV one can come you check and find that he has HIV but after telling him/her that you’re going to start medication tells you “I will not take it.” You ask why? “The tablet is too big.” I am just giving like an idea can’t you change that tablet may be reducing the size? Some people fail to start medication saying that the tablet is too big. Secondly some people especially women when you check her and she’s positive, you start her on medication but when she takes it home puts it in a place that is not right. Can put it in the kitchen or toilet; what she is avoiding is the husband to see it. The man comes with the medicine to the health facility to inquire. When you ask where did you find this tablet “I think it’s my wife using it but what does it do.” it’s what has brought me here you tell him that first go back and bring your wife. He goes when he is not okay now that’s why we ask “isn’t there any way you can change the voice to reduce the size of that tablet so that some can use it. May be puts it ina banana so that can swallow it. Some say “I will smash then swallow all that is problems.

F2- Your voice is very important especially about that tablet it big size and so many things right now the research around says that if we can get a pill because for it, it’s easy to swallow. There is some medicine doctors call long acting. You swallow and take every day, or swallow once a month, sometimes six times. But to force that onto us the project will have ended and even us, we have started getting grey hair so there those we can achieve later because for the project it has already started moving. That thing of investigating about that tablet if am guessing we might not achieve it now because pharmaceutical companies look at it has so many things. So this project cannot handle and another thing there is what they call real life if the project is successful that’s why I liked this lady’s when she said we train those very people instead of bringing others. You remember where we came from TASO committee all of them left with…. When USAID went and others came. Treatment regime for the country if we are still on the tablet it is what we have go with the project will not bring something new because there is no… there we have left real life. To summarize what I have said we have to do it in real life because the project started already I don’t think we can achieve that issue of changing medicine in these years it has to be there, we have eighteen months.

F1- He has elaborated well because that issue of medicine even DHO cannot change it.

F3- Another thing there are so many trainings so we don’t want to repeat what others have said. MRC is a huge project they try to teach so many things but that one is not this and they finished working but protocols are many…..

P- There is something I forgot to tell at you at first I want you to differentiate for me, Stress and depression.

F1- Now depression is “okwenyamira” it’s the Luganda word that we have, stress is situation that mistreats one psychologically but depression can come from stress. Stress can bring you depression eventually, depression comes from stress but stress has an element of worrying and unstable mind, for example you’re worried about fees and you feel your heart beats.

P- Stress is usually short lived like you can be there and the boss doesn’t pay you well or husband is harassing you. It’s there for a short while but depression is a long term condition. It will have been there for a month I think

F1- Two weeks

F3- Two weeks when you’re there in that situation some people withdraw from others , be sleeping, fail even to eat food because of that situation until… whereas stress something can come disturb you a bit but for prolonged stress can become depression.

F1- Have you had a loan and you don’t pay in time they call you all the time? Where is the money, do you remember what you went through? It’s an example of stress it worries you until you settle it but if you don’t it affects you; it can bring depression or other problems.

F2- It’s good in this project Professor said we shall train people like in such a session we’ll get lost if we start teaching about depression and stress. But there is something called mhGAP he said we shall get it and we teach people.

F1- Teaching them are the activity; sensitization what and what that need to be taught.

F2- I think this is good thing for a meeting what will work and what will not work

P- I was saying that our clients that have HIV they should bring them trainings for developing themselves because of this poverty.

P- They get stress and stress brings them depression.

F1- What you have said is important because it’s true what brings depression sometimes is poverty and even if they give him/her medicine goes back home…

P- When there is nothing to eat?

F1- But it can be above us we have to at least identify it like we have said later we… as we take out resources we see how they can be helped. All programs of TASO, that is how they started that project giving porridge and food. The problem we usually get is that sometimes people become lame and think that TASO is going to be providing me forever, but they are helping you for a while. That element of empowerment we talked about; we talk about self-help people need to be empowered so that they can get themselves out of that situation.

F2- Now like you hear this word HIV patient now this program we no longer use it if you go to TASO and say patients, they can even beat you. Now for that you have said it needs also them not to feel that they are sick.

P- What do they call themselves?

F1- Clients, they use those services because any person can be a client.

P - Mr. Ssebunya you told us this study has ever been done in India, how did they do it?

F1- I told you that they call it MANAS project. Somehow resembles this but it was not for HIV patients, it was to see that depression and anxiety are integrated and managed in primary health care setting. People who are there are, not psychiatric nurses like you but the general health workers… what will they do to see that they can screen people who have depression and manage it. And later, there are workshops we shall have…call it Health Activity Program, trying to show you how these depression patients are managed, how are they identified? How were they managed that if one is at this level is managed like this… So it was not specifically for HIV patients but it was for management of depression and anxiety in PHC setting.. Already we don’t need any separate program for managing depression in PHC because mental health is already integrated in PHC and in all districts most health workers have got some training for mental health care.

F3- Why are we just giving outcomes, I think we should go back to impact?

P - Teaching people

F1- There we go back behind so if you bring an idea and we have it already we are not going to write it twice I think we wrote that somewhere.

P- That’s capacity building

F1- Aaaahaa capacity building is sensitization

P- Training health workers

F1- To teach which people, all patients or…?

P- All of them

F1- Community sensitization we have district health education program. What else how do we reach that situation where there is no discrimination?

P- More emphasizing counseling of positive clients

F1- Counseling positive clients

P- With a positive living I think we talked about it on empowerment.

F1- Positive living what else; should bring the point it can be outcome when it’s not activity. We are asking ourselves what should be done so that we have a service that has no discrimination.

F3- Community sensitization on HIV or depression

F1- Of course community sensitization will cover both because we are not going to address depression in HIV and then sensitization we talk about HIV depression component has to feature.

P- I don’t know how we are going to put it some may be long term like you have said mainly will talk about government setting/facility setting. They know that building that it’s for those coming to get HIV drugs that everyone most cases it’s without patients. So when one looks and sees the other going to get tablets that stigmatizes them.

F1- You think there is labeled that HIV get from there, it brings stigma

P- Yeah

F1- But that stigma isn’t there also self-stigma to begin with? Why do I think people see me badly because I have branched here to get medicine? Okay that means we should have health facilities without any labeling and when HIV person comes and doesn’t know where to get medicine

F2- I think in our HIV campaign, there is one thing we have still failed to get. If you are in the village and one says “you know what, am HIV positive and on medication”… even these VHTs you say that they talking about people they cannot talk about that one because there is nothing new they will be saying.. That is what you said that may be if we empower them to overcome stigma they undertake to do self-disclosure. There is nothing new you’re saying for him he declares it.

P- There is this also let me tell you. I know that I have HIV and even in the village where I stay they know that I have HIV. Now when I get some disagreement with you, is that what you have to start with to abuse me?

F2- But if he tells you that and everyone knows and it’s you who said it doesn’t mean anything.

P- People quarrel even at work I have where am working may be teacher and we are there may be I have a mistake I have made, do you have to start with that?

F1- Sister, tell me, if we know that you have HIV and then I abuse you that you have HIV, of the two, who is stupid?

P- That comes later because of anger.

F1- Yes anger because anger makes you do a lot.

P- Yes by the time I think that you who has said that you’re stupid, I will have got angry already.

F2- Us in counseling, sister all those things concerning you show that you are not yet empowered...In the field of stigma there is what you have to know in overcoming stigma like accepting that you’re not like others. If you think they will treat you like others are, there you’re also wrong. If you have not reached that level of empowerment…, you have to accept that I am not that. What you’re now is what you have to accept you are not like others, if you fight with equality with those that have no disadvantage then you’re not also empowered. It cannot happen in real life even you when you have a patient mental case, you may say “at least am better”

P- You have discriminated

F2- So we have to accept that I have been seeing this program I was evaluating in Villa maria I have told you this thing of disclosure it’s about family because the children keep the medicine at home bring for daddy with water. Daddy you forget so much, time for medicine has reached and they bring for him and he swallows. That is what you’ve said…like malaria clinic, it means that he has to widen beyond the family everyone gets to know that he swallows medicine. So if the children take to him in the garden or at the butcher they just take him and he swallows so sister you have not reached there yet.

F1- The way life is if they get that support that they need but we cannot fight stigma from other people. Sometimes we have to think of perceived stigma but when it’s not there and we think others will laugh at us.

F2- Joshua we have to remember when we were starting VHT campaign, we used to say qualities of a VHT; confidentiality do you remember them? VHT it’s now a job there is something coming and VHT became a stepping stone to become LC. So, that became a job. We should not emphasize that word VHT because us who work in PHC we know it doesn’t work anymore, you will check yourself we shall use either expert client or VHT. It’s you to advise us on that but I have told you VHT became a job and politics.

P- There are those who work

F1- Okay to get improved health outcome among these people who have HIV to see that they are well, what are we supposed to do? What is supposed to be there even if it’s not who have done them? Those that came make people with HIV and depression to be well.

P- To be with communication

F1- How?

P- Someone who has HIV has to be positive being in distance you have to have communication with him/her

F1- Who?

P- Okay like expert client and that person

F1- You are talking of effective communication between expert clients and client but I would say between the clients and the system. System it can be VHT, expert client and even doctor through any other person because the doctors have to know what is going on and the people they treated; how are they?

P- Because in communication we are trying to know how the person is

P- I hope like we said that there is a situation where one can reach and is depressed and it may require treatment. So if it needs treatment let the medicine be there.

F1- Resources input and drugs; I did not write that actually this one have put it far but it should be an outcome. So we were looking at communication between the patients and the system it’s very important it has to be there another thing drugs we have talked about it.

P- The first thing I think is screening

F1- Screening we finished that already, what other condition has to be there so that these people can be well

P- The doctors and training

F1- Even doctors have to be there

p- They can be there but when doing nothing

P- It’s true

F1- There some districts when staffing levels are as low as 50% so if you tell me that health centre they have like two.

P - Training we put that already.

P- Availability of other resources.

F1- Which resources?

P- Drugs.

F1- Medicine is available which other resources do we need?

P- Stationary.

P- Tools.

F3- Tools may be.

F1- Which tools?

P- Assessment tools.

F1- Assessment/screening tools

F2- Let me teach you something, do you have rooms here because what I saw in Gulu? Four patients everyone has a doctor but talking different conversation. Do you have rooms?

P- There was no privacy at all

F2- Do you have rooms here?

P- We have enough rooms at least

P- We are talking of space

F2- What about food? I was seeing in Masaka that what make people refuse to swallow this medicine is eats.

P- Its true there those who don’t swallow medicine

P- But we have been on this issue of HIV and health workers, not so?

F1- Yes

F2- We said we are talking about every thing

F1- If the point has come

P- Eats are not there to some of them

F1- Let me ask as an illustration for example what can show that we have improved health outcomes. The indicators, what indicators will show that service has improved? Or people have improved, how will we know?

P- Like for depression for us to know that the program is picking up we shall look at how many have we screened? How many were supposed to be screened then out of those; how many are screened? Out of those who are screened, how many have you seen depressed and if they are depressed are they managed? What’s the outcome?

F1- These alone could be enough if you give me report on number you have screened and the ones you managed and the way you managed them, I may not ask for outcome because you may not show that. You can say this one I managed this one with depression except when you stay with him/her that he was like this now is like this. Especially health worker if that was stoke, you cannot say I managed that one and the outcome is here so in this case would be an indicator. This clinic before we did not mind but now we screen this number of people last month we screened this number for depression and managed them like this and this month we have managed them like this. Eventually you see numbers there is change of course we shall not hope that data entry will be there. If I see the same people every month you screen, there I just know you’re lying but if you show me that last month we screened 60 and had 15 who are depressed and managed them like this; this month we had 50 we found 6 and managed them the trend can show you. So reports on numbers could be an indicator we said services and resources for reporting depression are available at the facility. How will we know that service and resources are available at the facility? Because we are starting but facilities will not pick at the same time, how will we know at the facility services are available and are working?

P- We can use patient’s files.

P- And register

P- We can create a column in any register.

F1- Of course we shall do research

F2- But in natural environment when I look at the patients that come and those that have been worked on, it is an indicator.

F1- If we identify well the outcomes and activities because we shall delay these indicators are easy they don’t need scratching the head. We shall know form training how is an indicator developed? Number of patients how is it achieved but because training capacity building probably training were conducted, what will show that these people there capacity was built? Is it the training that once we do training then we say we built capacity?

P- We see the number of clients screened and assessed is it increasing or not?

F2- Okay that is one of the indicators, what other indicator can show that capacity of health workers was built?

P- What I see there is that what you taught us have you found on ground when are doing them?

F1- How are you going to test them?

P- Because I can screen without documenting, when you come, I will tell you that I screen but what shows that I screen. So if we talk about screening it should be screening and documenting that you have screened.

F2- If the doctor is working what happens?

P- How many patients have cured or dead

P- Number of patients screened and found with depression then those one who recovered.

F1- Yes those that recovered, how will we know that they recovered?

P- When there are no manifestation of signs and symptoms of depression because if I start on him, it means that there is presentation? Those things he shows if they reduce and he doesn’t have them completely, if I screen and see does not score the level is not depressed it means he has recovering.

F1- Now if he/she has not understood the dose of course you the one treating you so he /she for one day so when he comes back you get to know that you gave him under dose. If the treatment was counseling dose I gave in that counseling was not enough. Any way you can know that I need to change may be there those I did not address well or didn’t do well.

P- This counseling starts on drugs because this medicine it may work but when the person has been sat down for counseling.

F1- Now the truth is most depression patients don’t need medicine, counseling helps them a lot.

P- When they are sick and don’t need medicine

F1- I didn’t say they don’t need but most of them we can say 52% to 100%. So depending on what has brought the depression because if you’re depressed because you’re scared, the medicine is not going to change the thoughts to go. There is no medicine that takes away thoughts, how they came is the way they have to go. It’s not HIV that brings them there is no virus that brings thoughts therefore even medicine can’t take them. All illnesses that are psychological the treatment that works most still has to be psychological even though it helps sometimes but the other is better.

F3- What I wanted to say is that treatment or guidelines for depression are there you will be taught…. that workshop is there. Like Joshua has said whatever treatment patient is given depending on the condition will be it has to have counseling.

P- Of course there is what we talked about financial and what to eat for our clients. We have community development organizations with us that have been trying where we refer some of our clients. Some they empower them to work as them, some who have children, is it social support? those that don’t go to school they take them but not for good but sponsor your child like for three years as they also empower you to learn how to make money. You start paying for the child so I think they can help in such a situation.

F1- if we link them to PCOs

P- They can help so that it is not upon the facility but lay patients can get service elsewhere if he/she knows s where it is.

P- I would think that biggest stakeholder is government because this program can end tomorrow but how will it go forward, the government has to involve its self.

F1- But also government is involved. It is waiting for findings. That is why we have been with Mr. Taasi the first meetings we had; that is the HIV focal person at the Ministry headquarters. So they are waiting to see this program; how it is going to inform national strategy so that they incorporate it.

F2- There is full recovery what if we say symptoms because saying full recovery….

F1- Full recovery from depression

F2- Because we know there are others

F1- Yeah but when depression goes it doesn’t mean malaria or TB will go too.

F2- Its full recovery from depression maybe we can leave it

F1- I think let’s just keep it around depression. I said one of the things that we need to know here it will come under activities because at health facility, when we involved health workers we need to know causes and factors of depression among people with HIV. We hinted on that but can we elaborate more? It may not fit in TOC workshop fully but it’s important in planning interventions in this workshop. The health workers or counselors when teaching have to know what commonly brings depression then know how to handle. If I don’t know why people with HIV usually are depressed, it will be difficult to help them because I will put my theory from class and yet there it doesn’t work. What commonly brings depression among HIV patients?

P- The fact that they are taking ARVs for life that alone can depress him/her having known that is going to take medicine all his/her life.

F1- His/her whole life that automatically may make him depressed. Okay the fact that they have to be on medication throughout life.

P- The stigma

F1- The stigma because these are the key issues the health workers will have to address.

P- Poverty

F1- Poverty that one we talked about because there so many things in it

P- Other stressors like domestic violence, famine

F1- Are there things limited to people HIV apart from being on medicine for life but poverty, stigma even us they concern us.

F3- Frequent hospitalization

P- There is something I have seen actually this program will help us, I had never noticed that there people who have been falling victims of depression but failed to discover, how? There are clients who left medicines for long time when they come for clearance you see that they are okay but when you do viral load it turns the other way round. That thing gradual process you see him/her loosing falling into depression yeah, I have just seen that now, I don’t know if it’s a cause that .

F1- You want to say that depression is common among patients even though is declaring

P- When results come, can tell you that is swallowing medicine but when results come it’s that virus is none suppressed however much has taken long on ART.

F1- Because immune system is compromised, has stress, depression which is not addressed so those things weaken your immunity.

P- Actually the point is for them to think they are taking well because he has spent long time but in the system. We shall look at it in frequency but what I am saying when you do baseline viral load and you tell him/her that it shows it depressed

F1- Could it be that is taking medicine but failed to cope; accepted to take medicine because he/she has to take it but has never accepted the situation?

P- Sometimes they’re being burdened with drug side effects

F1- Side effects of the drugs, does it affect us so much up to now

P- Yes they’re there

F1- Side effects

P- When the tablets are many

F1- It’s very good when all these things come out because some health workers have to know and go through them. This client before you, what might be some of the thing is experiencing because they lead them into depression it’s what we have to address.

P- The irregularities in the chain of drug distribution that if someone is not certain that the medicine is going to be there.

F1- These days I hear on radio but I don’t understand it that there are some districts you go and say “we have a lot of ARVs that we can’t even finish.

P- It’s not there.

F1- In fact I was in Kamuli recently and that is what I heard.

P- It can be there for certain regiments.

P- This time yes it’s true some regiments may be and others are not there.

F2- We have focused so much on demand side but system…..

F- You said irregularities of supply of drugs why don’t we say some?

P- Can we say some ARVs.

F- Because we said some are there out of sixteen only one is there.

F1- We better leave it because we can’t have a perfect system where all medicines are readily available, we have captured it, but it’s beyond us. Health workers how do you address that? There is patient before you, is worried that what if the medicine is not there. How sure are you when it’s not even you to buy it?

P- Of course you give constant assurance because you say “we shall give you for two weeks but have hope that medicine is coming” you keep assuring them.

F1- And it’s not us who are going to bring it for depression

P- You’re not going to bring?

F1- Anti-depressants… it’s not us to bring, the truth is most health centers you have anti-depressants but you don’t use them and others even use them wrongly. Mental medicines are there some are used wrongly others are not used and they expire. So it’s just adjusting on our budget that which medicines do we need most? Because even if the budget doesn’t change, we can still change or that facility needs this and us we don’t use it

P- Let me assure you it has ever been done, MNS supplies only Amitriptyline but fluoxetine

F2- You’re supposed to order for them.

P- Yes we order for them I went to the pharmacist we put there but at the end of the day they give you Amitriptyline. Those other medicines were there during the study by Mildmay, but when ended, all the clients that remained on it…. we put an order but they just brought Amitriptyline.

P- I am also adding on what you said that such matters are beyond us and the way we use… that study was also in Buwama, Mpigi didn’t have Fluoxetine. We used to send them because we had it in Buwama but it expired; we were not using it.

P- The thing is you had it and even if you had given it to me, would I get the continuous supply of fluoxetine?

F1- Okay this is beyond us or even DHO but you can do something about it, isn’t there a focal person for mental health in the district?

P- He is the one

P- Am the one

F1- Okay you’re the one why is it that in facility X medicines expire and yet facility Y needs it? Much as MNS has its weaknesses, it really tries to avail medicines. We are hopeful that the PUSH system is going to end…. but in the meantime, districts themselves can improve, can have internal arrangements that help them. What MNS fears most is the budget. You can always make some adjustments as long as you’re within your budget.

F2- What I have seen the problem is how do you convince MNS to send you when even the little you have is expiring? The problems come from what the lady said that you don’t record medicine because when one comes, this thing of mental health you don’t look at it even DHO told us that. So if we create evidence that it is needed and the numbers are there for those you’re treating, you can have evidence even to approach the Ministry. I got surprised when I was working in Gulu in another project like this one, I first bought but I realized I was wasting time buying medicine yet at some facility it was there expiring; because there was no psychiatric nurse. I had put psychiatric nurse. So, the problem is you don’t write.

P- Some we write like for epilepsy

F1- Phenytoin

P- It’s there

P- We don’t write sincerely speaking

F1- Now apart from writing not only medicine but also your HMIS reports. How do they look like? That medicine you ask for and the patients you report it in HMIS reports… You know how you prepare HMIS reports. That also affects planning. If you start demanding for medicine X, let your medical reports show that those patients are there. But unfortunately, in most cases the demand for the medicine is not reflected in your HMIS reports. And the system can’t be supportive when Of course in the trainings, we shall need to address that, because it gets beyond us. But DHO and focal persons who are responsible for that are there. Sometimes we blame others but when even us don not do work well.

F2- It’s not only here is Uganda but globally when they are manufacturing medicines they depend on disease burden in that; there two things that matter mortality and morbidity. Those who have died of that condition and those who have contracted it, isn’t that so?

All- Yes

F2- Now how do we prove the disease burden when we have not written in HMIS because if you write, they go into HMIS. If you don’t write they don’t go in HMIS. For finance they cannot give you medicine on a disease that is not there. It is us who create that situation that the disease is not there.

F1- You create malaria that isn’t there every patients is given coartem,

P- It is no longer happening in Mpigi

F1- But it used to be because malaria was common so they knew that coartem is what you need most. So with time if we improve our reporting system even the drug system will improve.

F2- The spirit of theory of change is that we identify problems and suggest the solutions that is why I said that if we emphasize psychotherapy so that one doesn’t go with nothing. You also imagine someone comes riding his bicycle and you tell him he has no problem at all. He will think there is nothing you have done for him/her.

F1- I don’t know if the health workers have heard that because at least one goes back saying “the doctor gave me 20 minutes and talked to me.” But these conditions we talking about depression the majority of the patients will benefit from psychotherapy more than anti-depressants. Therefore we need less anti- depressants but we need more psychotherapy; counseling what we are trying to address here.

P- Now there is a problem may be they add manpower

P- That is it. You are seated this patient comes with a problem when you look outside and the triage… the line is long.

P- Even the patients themselves quarrel why has that one delayed there?

F1- And that is your internal arrangement; does it make sense anymore, screening them on one day and yet we have to address their issues?

P- Now in Mpigi, it’s a full week

F1- Everyday?

P- Yes, up to Friday

P- Yes there is pediatric, adolescent and FSG though they cannot be so many everyday but ART clinic day will have like 100 plus patients a day.

F1- So what do you think should be done?

P- Because there are no specific clinicians for them…. they keep on rotating, OPD and general ward. some will go to OPD and also works on general ward ,the one remaining will come and help me in ART clinic one or two but now days.

F1- Let’s be realistic. What are we going to do to prevent this depression we need extra time?

P- We need manpower

P- In addition to manpower, there are those things that are periodic you may find that even our place we see many but why does it come like that? You find that supply like we said when the system has a gap it can affect everything going on. You find that those you have been giving for two months they go and you know that in those two months you will be able to see a few, you will give them a monthly refill which means the turn over at the facility will increase. That will be affecting the service but what has brought it is less supply that in case you get enough supplies you can be able to distribute on various days.

F1- I thought health workers that run the HIV clinic don’t often get transferred.

F4- They don’t change but they multitask… they work elsewhere

P- When a client enters in the room I want to give her/him time but there is a line and others are quarreling, why has that one delayed? What are they telling her? To get someone like we had identified comes to the clinical room we do his/her checks well then tell him/her that you will go to the other room like we do on viral load. We get their books and leave the book at the window take the paper behind they test blood or when you leave the window with this file we are going to see the counselor or IC then see the counselor.

P- But who is going to write the medicine?

F2- We have said you wrote first now goes to expert patient.

P- How did we do it with doctor Akena?

P- We had two questions but when you go to the clinical room they are nine questions all that is time. Asking this one person all those questions I would think that this person is where they would train someone to ask those questions.

F2- Those nine questions if you have experience in counseling they are easy in the process when you get the form its part of the process if you have experience

P- Now what we did before when they came with two questions not everyone who was asked those two questions qualified. They would enter but when the forms are on the table, we used to write in the books this one has scored this but the problem we want to talk about this person needs time. I can do this in the clinical room where I am but what of those that need more time. Why don’t I ask these questions in the clinical room write and send him to you in other room?

P- Me I think that person who has come with such a problem especially when the clinician needs to counsel should be given to him so that he can see him. But there are those that can come without any problem because what I see at the health center even that one without a problem is also sent to the clinician’s room. That one who has a problem even if you say that you’re going to solve it, the clients outside are quarreling why has he delayed but us we were suggesting that at least the clinicians are given people with problems that need them. Those others for refill even if you don’t have anything paining you just came to get medicine… other people can see them.

END
