# Supplementary material for: Using Theory of Change to inform the design of the HIV+D intervention for integrating the management of depression in routine HIV care in Uganda
Source: PLoS One. 2021 Nov 30;16(11):e0259425. doi: 10.1371/journal.pone.0259425 (PMC8631669; doi:10.1371/journal.pone.0259425)
Supplement: S3 File — (DOC) [file pone.0259425.s004.doc]

**Third ToC Workshop held in Mpigi on 23^rd^ February 2018**

**Attendees**

- MRC staff
- Chief Administrative Officer (CAO)
- Senior Community Development Officer (SCDO)
- Health workers – Nursing officers
- Counsellors
- People Living With HIV/AIDs (PLWHA) representatives
- Village Health Team (VHT) representatives
- Expert clients

**Key**

1. Professor Eugene (E) 3. James (J) 5. DHO (R1)
2. Dr. Maggie (M) 4. Joshua (F) 6. CAO (R2)

E: Thanks very much for that prayer, I think in order for us to discuss more comfortably, let us get to know each other. Let us do some introductions. Let us start with madam here;

1. NF – Senior Nursing Officer, Mpigi Health Centre IV,
2. NR – In charge Butoro HCIII,
3. CK – standing in for Charles Kagwa, TB Focal Person at the same time working with Kagwa as an assistant,
4. ND – coordinator, People Living With HIV(PLWHA) through Mpigi PHA forum,
5. SM – Counsellor, Nkozzi,
6. MNL – Senior Community Development Officer in charge of Youth, Culture and Gender,
7. JB – VHT, Mpigi HCIV Mpigi town council,
8. AB – Elma Counselor, Mpigi HCIV,
9. SS – Expert Client, Mpigi HCIV,
10. BD – VHT, Mpigi HCIV,
11. Richard Ssali – District Health Educator standing in for the DEO,
12. AK – District Development Officer, Health Focal Person
13. Other respondents in the group (R)

E: After those brief introductions, I think now we are comfortable, we can have an open discussion, so let me give you just a few brief remarks on why we are having this meeting. So the reason why we are having this meeting is that we have so far had... I think three previous meetings in this place. The first meeting is when we launched this study, the second meeting is when we were discussing how we can bring depression management into HIV care. So for that one we have had two meetings. We had a meeting where we had the health managers in the meeting, then we also had a second meeting where we had the clinicians and the community health workers and that is maybe the expert patients and the counsellors. So now, our expert Mr. Joshua Ssebunya has put all these things together, he now wants to present the map that we have developed through the discussions we have had so that you can also give feedback and say, no, we did not say that one actually we said this. So he is going to have an opportunity to present and then we shall also discuss with him and give him feedback and say no, no, this I think we can modify this map here, we can leave things a bit better here, so that is why we have called for this meeting. But before we begin this, because now you are attending this meeting as research participants. Your views are going to be used to enrich this map. So I am going to call upon Dr. Margret Nampijja to take us through the consent process because we must get permission to record your voices and get your ideas. So without wasting so much time, let me call Dr. Magret Nampijja to take us through the consent process.

M: Good morning every one, like professor said you are participants and therefore we need to show that you have consented, you have not just been thrown onto something. So each one of you has two consent forms. The health workers should have one that has the word health workers, the non-health workers should have one that has non health workers. It can be English or Luganda, so you choose your preference as you are signing in. So there are two copies of the same thing (*consent form*) for each person.

E: Maybe let us decide, what language are we going to use?

M: Yes, that is important.

E: Maybe do we all understand English? Who are those that understand English? I mean who are comfortable with English? All of us? What of those who are not comfortable with English?

M: Someone whispered they understand Luganda better. I think I will mix and mingle.

F: I think it should be easy…

M: I will do it in Luganda. Anyone who does not understand Luganda?

We are here to… like professor said to do this activity, the final ToC or the final Theory of Change workshop to review the map, so just like the previous workshops that we have had, I am sure we, at least each one of you had one chance to be in the previous workshops we have had. So this is exactly the same consent form because it is about the same focus, the same activity/objective. So just to take you again, to remind you about what this information is about. The first page tells us about this whole project and we know that 30% of people living with HIV have depression and that is defined in long sentences; feeling week, you are of no importance/hopeless, you do not get good sleep, such symptoms are what we call depression. So what happened is, the Uganda National HIV /AIDs strategic plan recommended that depression care be integrated into HIV care for this reason, because we have like every three people, one person has depression among those who have HIV. So in response to that call, MRC led by Professor Eugene developed this project in which they want to…we want to develop and evaluate a model for integration of depression management into routine HIV care. When you do not understand anything, please put up your hand quickly and I clarify. So to explain more about the HIV+D project, this is based on the MANAS intervention from India, which was a project or system that was working in India and we thought that it would work here in Uganda. Of course it had to go through this system of trying it out and that is where we are up to right now.

E: Maybe to add on that, we are going to have even another set of workshops apart from this one. This set of workshops is going to help us to see, where we are going to put… I mean where are the different components of depression management going to take place? Shall we have some components in the community? Are we going to have for example the screening for people with depression in the community, is it going to be in the health centre? Are the people in the community going to play some role or another?

But after we have done this, (*referring to the workshop*) actually there is a lady who is coming on the 19^th^ and we are now going to get the very treatment – the counselling. We are going to call you back again and she is going to present a number of treatment options that are there and we shall discuss with you people and say okay, this component – I think we can manage, this one I do not think it is applicable here. So, this is just discussing about the structure. How are we going to structure the treatment? After that, we are going to discuss the very treatment itself – the very different methods of treatment. It could be the next phase after we have discussed where we are going to do the different parts of care; where are we going to do the screening? Where are we going to do the treatment? Where are we going to do the referrals? is what we are discussing in this set of three workshops.

M: Thank you. The next question at the bottom of the page, Luganda/ English; why have I been chosen to take part in this study? You have been asked to participate in this study because you are a health worker or a health manager involved in HIV care and Mental health and for those who are not health workers, there is also a reason that you are either an expert patient or you are from an NGO representing, I mean working with people with HIV delivering services for HIV care or you are kind of related – you are not a health worker but…

E: Could be Village Health Team,

M: Yes you are a VHT,

E: Or a counsellor,

M: So in that respect, we would really benefit a lot from your ideas- from your contributions to these workshops. So what will happen if I take part? If you accept to take part, we are going to have workshops (already we have had workshops like Launch, HIV+D1 workshop and second and this is the third one) and as Professor has explained that we are going to have more activities (*workshops*) ahead with the same purpose to evaluate how this project will be run in the different units or areas/organisations you are coming from. So your ideas will be very, very welcome. We had mentioned a total of 4 meetings but as we go along, we may invite you for other activities as well but for each one of them, we shall be explaining to you the purpose and ask for your permission officially just like we are doing now.

What happens if I refuse to participate? Participation in this study is completely voluntary and if you decide not to take part, it will not affect your job or receiving any services that you have been receiving and if you refuse to take part in this study, it will not affect your ability to take part in any other aspects of the work now, or in the future. If you agree to take part, you may later withdraw at any time and without giving a reason. If you decide not to take part in this study, it will not stop you from participating in other studies.

What are the risks and benefits of my participation? Risks are usually bad things. There are no major risks – maybe what you need to know is that we are going to be here for some time (it may be up to lunch or even come back after lunch to fully discuss this) for us to put this together thus obtain the best out of it which we shall include in our intervention of HIV and depression management.

Will there be any costs or payments? Just like how we began, this is voluntary for the benefit of our country and therefore no any form of payment apart from reimbursement for your transport that you used to come here otherwise there is no salary or any payment for participating in this study.

What happens to the information I give? Your contributions in these workshops will be audio recorded, transcribed and subject to qualitative analysis to provide information that will form the local adaption of the MANAS to the HIV situation in Uganda. The results will be written up and then presented to people in charge of health services and they will also be published in research journals or in presentations, videos, displays, posters and web resources.

Confidentiality – though I have said we are going to audio record or video record, the information received will be confidential and that is the point we are on. All the information given by you will be kept confidential and only staff will have access to this information, responses will be anonymised so that names will not be linked to responses. All information will be stored in a secure manner and will not be identified by the records that we keep. So that is confidentiality.

So what happens now? What is going to happen in these workshops is that we are going to be brainstorming and whoever has an idea on how you want this to move, we request for your opinions. So, so far we have had 2 workshops plus the HIV+D Launch and all that is a process that we are going through. So this is another workshop like the kinds that have happened so we really welcome your input – your contributions to this.

Who Can I contact for more information? (*On HIV+D project*) The Principal investigator for this project is Professor Eugine Kinyanda who is just from speaking to us – a senior psychiatrist based at MRC Entebbe. He has experience in Mental Health and HIV research and you can contact him on phone number…each one of you has that phone number indicated for Professor Eugine and if you would like to discuss the study further or have questions, suggestions or complaints then please feel free to speak with any of the study team.

The study is funded by WELCOME TRUST, so if you need to know anything from the real boss – Professor Eugine, you can contact him on that number or if it is general you can contact the staff (they identified themselves). Am I clear so far? Are we all together?

R: (All participants responded, yes)

M: Who has approved this study to take place?

We did not just wake up to bring it to you, it went through steps, reviews and was accepted. So the study has been approved by Research Ethics Committee and UVR Institute and National Council for Science and Technology. They have looked at this work carefully and they have approved that this is important work and that the participants’ rights and safety have been respected according to what professor and his team wrote in that proposal – so it was approved.

What if I have any questions? Just like I said if you have any other questions or need clarification you can contact Professor Eugine himself on that number or Dr. Nampijja (myself) on that number or Dr. Richard on that number. I mean these are the key people besides the head, then two key project leaders but anyone on the team, you can reach them and ask them about it. On the phones these are the phones that are available.

What if you want to ask someone independent about anything about this research or any questions about our rights as research participant? (You might wonder we are the same people on research at the same time explain to you about this) You may speak to the ethics committee chair person from Uganda Virus Research Institute. There is someone in charge of all the research at UVRI, he’s called Dr. Lutalo (0414321962) you can call on it and he is fully aware of it (*HIV+D research project*).

So that is the information or explanation about this study and the activities you are going to be engaged in or those you have already engaged in because I believe that most of you have been part/present in the previous workshops.

Before I get to the page for signing, I request anyone with a question to ask, if not a question, any comment – most especially I would like to know if we have understood. I request to ask one question randomly to evaluate if you have understood what I have explained to you. I could have explained but you have understood differently/in a different way. So yes… I will ask one gentleman and one lady.

We have said that the purpose of these activities is what? What is it we want to do? What is the purpose?

R: The purpose is to integrate depression into HIV care.

R: (All clapping)

M: Yes I hope we all have the same answer. If you have that answer that means you have understood.

I have another question but I request a male to answer it; we said that accepting to participate in these workshops or activities, is it a must to participate or no?

R: Participation in this study is voluntary. You have the right to accept or pull out.

R: (All clapping)

M: One more question (I should have brought first) so if we have been asked to participate in the study and we accept what is expected of us who have come to participate (I think I give a chance to another gentleman) like we have come today, what do you think we need to do?

R: As you said, when we are here, we have to brainstorm, discuss broadly on how we can do this project.

R: (All clapping)

M: Another clap; so thank you very much, the representatives show that everyone has understood. I cannot ask everything but those few questions really show that we have understood.

So we go to the last page; the page like this one (shows to them the page) which has some blank spaces, it is the one we call the consent form. So this consent form has parts – the top bit, from the title then you go to name of the participant – health workers in brackets non health workers. If you are a health worker, you should have health worker, if you are non-health worker, you should have non health worker, then the name of the health centre where you come from whether a hospital/ health centre or organisation, the job title – you may be an expert client, VHT, CAO or Assistant CAO, so please your titles and then please tick if agreed. We have 6 boxes which have information and you tick if you agree to the information. The first one on top is; I have read and understood the information that describes the study or I have fully understood the explanation of the study offered, I understand what will be required from me if I take part in it and my participation is voluntary. Then the second one; I understand that, at any time I may withdraw from this study without giving reason and without affecting my work or normal care or my participation in any other studies to which I may want to take part. The third bullet; I consent to the processing of my personal information for the purpose of this research study and understand that such information will be treated strictly as confidential. Fourth; I agree to take part in the discussions and the meetings and workshops held in the study. Fifth; I agree to participate in in-depth individual interview involved in this study- we have not yet started on that but in the future it may happen. You can tick just in case we happen to have it. Finally, the interviews, discussions, trainings or sessions to which I may participate will be video or audio recorded and photographed and used in presentations, displays, posters and web resources. So those boxes you tick as you agree.

So my questions concerning this study have been answered to my satisfaction. I asked you and I hope you understood. So anyone who has questions please ask.

Then after that, there is a free line, my signature, and thumbprint. Below indicates that I agree to take part in this study so you sign and put the date on this line which has signature, date, and those who are unable to sign, maybe they have difficulties they cannot write their name, you can put a thumbprint in this box. So do not sign at the same time put a thumbprint – you do one of the two, is that understood? And if you decide to put a thumbprint, we shall get a witness to sign in this space under witness – witness name, signature and date. Then here, investigator will sign, that is, staff name, signature and date. As for you, you need to sign is this top part; the signature and date or thumbprint if you are unable to sign. Still up, from the start, name of participant – you write your name, name of health centre – you write job title as I told you. ID numbers you can leave them out – they are not necessary. So that is it, everyone has two copies, you sign both of them in original, keep one for yourself and the other one you leave it for us. Have we understood? You sign two copies, you keep one for yourself and one for us. Unless there is any other question. So we shall go round, we have done this before except for a few people who are representing others but most of you have been signing. Any question?

R: None.

M: We are going to start the process of signing and we will be there to support you.

E: Thank you Dr. Margret Nampijja for taking us through that session, let us give her a hand clap. So once we have finished the consent process, before we go into the actual digging of the garden, I think we need some energy; so once we finish consenting and have handed over your form to Margret, there is a cup of tea that is outside here then we shall be back here to dig into Joshua’s garden. Thank you.

F: It is good we have agreed that, just like the last time we shall be using both languages. We were here like my colleagues have told you, we held two workshops here and we got ideas from you. We do not have much time, I will try to use the time we have to be precise and get to the point but at the same time to make sure we are on the same page. So during those two workshops, we generated ideas from you towards this programme. We emphasised that this HIV+D is going to implement a programme in the district; but we are not coming in with preconceived ideas from our side but rather getting ideas from you people. I was saying if you remember, for those of you who were in this room, as we got ideas from you we even used some flipcharts, trying to summarise what we were giving. Then we had a second one in the other room down there – I think the sister there is among those who were there; again we got nice ideas from you. Now those ideas were contributing towards the roadmap that the project is going to take. We were identifying the activities to undertake, the outcomes we expect, etc. you remember we called it Theory of Change (ToC). And if I may remind you a bit, we told you that usually for programmes, especially health programmes, sometimes we sit there and think of interventions or activities to undertake without any sufficient background that we are basing on. But this time, we are talking of, you know, undertaking a programme to ensure that depression is managed in HIV care and you remember I told you we are using Theory of Change approach. We were starting with identifying what we want to achieve and not identifying what we want to do, but what we want to achieve and then we moved backwards, to get to this we should have got this; if we are to get this, this and that have to be there. For this to be achieved, we need this, this and that to be done. You remember that? Then we kept going backwards until we got to ‘what do we begin with, what has to be done first? I want to put emphasis, there are many programmes… here we started by identifying; what is it we want to achieve? What has to be available/present in the middle there? We kept going back to where we started from. What needs to be done first/ bottom (Wansi). Remember when we looked at the health system, we looked at three levels; those three levels are where much of the work is done in Health System. We have the Health Administration; that is the District Health Administration - DHO’s office and other offices at the administration; they have a role to play. We looked at facility level; there are things that have to happen at Health Facility Level – different Health Facility Level, we have HCIV, HCIII, the hospital, assuming you have one up to HCI. But HCI features more in the community. So we had to identify things at community level as well. So there are things that have to happen at Health Organization Level, the Health Facilities Level and then within the community. So we got a lot of ideas from you people. When I say a lot, some were repetitions, some were…, and so when we went away, we tried to go through all our discussions and extract all useful information and then put it together to come up with what we are calling a map – a Theory of Change Map. It is actually summarising our ideas but it is still a draft in a sense that there could be some things we still need to add or to remove. So the purpose of this activity today is to look at what we have so far. We are going to look at the ideas you gave us in the previous workshops. We were able to generate from them a Theory of Change Map which we have right now on the paper we are looking at. But it is still in a draft form much as we have tried to extract what we feel is important. It is still in draft and this third meeting we want to look at it and see; is it what we actually talked about? Of course we did not include everything as was said because if in the first meeting someone mentioned their view and in the second meeting one gives the same view; they may use different phrases/words but when it is the same idea. You might find that one point comprises of ideas from about six people talking about same thing. For example, if we say training, some people call it educating, others say orienting, others say building capacity, but all are referring to one thing that people need training to be effective. So you might wonder that ‘this is all we got from the two meetings’ no; but it is still in draft form and in this meeting we want to look at it and see was anything left out? Is there anything that was included that needs to be removed. Are there those that do not make sense? Is there anything we need to add? Is there something we need to adjust such that we can refine it? So we hope that by the time we leave this place, we shall go back and refine it. In doing this, we tried as much as possible not to include our views. We have not reached a stage for us to include our views. We are still depending on your ideas for you who accepted to participate in this. Of course we may also have ideas which are related to yours so when it comes to writing, we may have to adjust the grammar and make a few adjustments but most important our goal is to present your views much as the language may be adjusted. But as of now, even the language has not been adjusted we are still with your views. So we are going to go through them, we see where we need to adjust, where we need additions, etc. Please in case you do not understand, let me know and we go through.

I would like to take you through, fortunately each one of us has a copy, but some of us may have been looking at them but not yet understanding what’s there. I’m going to first take you through then after panel bit it together. Were all the views brought out well? Is there anything missing? Is there anything we need to add? Is there anything we need to change? As we shall see. Before that, I would like to remind you that when you look at the map, we have five or six key elements on the map. The very first one is the one we identified to begin with but it is also not finalised, but by the time we leave this meeting, we will have finalised on it. We were beginning with the impact. Impact is the long term goal we want to achieve (overall), which might be difficult to fully achieve but we are going to try to achieve it. I remember in the first meeting, we said, we are talking about HIV+D project which is focussing on integration of depression management into HIV care in the country – much as we have started in Mpigi but it shall spread because Ministry of Health is supporting this programme. Now what is the overall we want to achieve? The first meeting we held, the impact was derived right from the proposal and we said “Management of Depression integrated into HIV Care” – that we can leave that as the impact. I think we all agree that the impact should be broad. When we talk about impact, we mean, what is it that the programme is going to change on people’s lives and the community at large over time? It is not something you are going to achieve very fast. When you do something, there are results you are going to realise very fast/soon. There is where we have outputs, there is outcomes. Outcomes can be short term, intermediate or long term. The impact is long term and it takes time – we said impact can be long term outcome. But we are talking about something that will last and is evident, are you getting me? E.g. if there was war in a certain place, e.g. Japan war with bombings, up to now the impact of the war is evident. There are some people whose children are still affected due to effect on genes. That is what we call impact. The immediate outcome was that many people died but the impact is till pronounced even after many years – more than 70yrs. We are also talking about the impact that will remain pronounced much as the project will have ended and systems changed, but what the project will have introduced – its impact will remain. So currently we have three and we have to agree on one and when you look at the three, one or two of them, as we agree, could be modified, could be pushed into outcomes or maybe long term or intermediate. We can adjust accordingly and that is where we are going to start from. But maybe we can do that when we get to the discussion. I was saying this part is for taking you through. So we identified the impact. I said we have five or six key components in this. The impact, the outcomes that we expect – both intermediate and long term, we have indicators – things that will show that whatever we are talking about is actually happening. We have assumptions (we assume if this is done, this will be the outcome or for this to happen, this should be in place). Assumptions are those things we take for granted that they are available. If we do this, that will be the outcome but in the middle there is an assumption. Just as we look ahead and say if we train Health Workers and give them knowledge, they will work as expected – the assumption is they have interest. There is possibility that they can be given knowledge when they have no interest or you even call them for training and they do not turn up. So we have the assumption, then impact, indicators and outcome ... and we also identified interventions – what needs to be done to get to what we have been talking about. In our discussions, we identified inputs as well and key resource persons though this did not feature in the map. We tried to identify all that and we tried to summarize them here.

I will begin with the District Health Organisation level. At this level, from what we discussed in those/the previous two workshops, we have so far identified some 3 interventions (you remember I have said here we are going to agree, we shall add or remove some interventions). So far we have identified 3 interventions at the organisation level but they are not in the order; one of them is building capacity for monitoring supervision and follow up. Now this is capacity for the administration not the technical people – the Health Workers alone. We often think that capacity building is for health workers but we are talking capacity of the district health managers to monitor, supervise and follow up. We believe there is going to be a lot of focus here (Ekinyusi), because if the administration does not follow up what happen at the lower levels, most of the time we know what follows and we have seen this in most of the districts. We have often seen this in several districts and it arises from the ineffectiveness of the manager to supervise/follow up what needs to be done. E.g. in one district the manager who was supposed to supervise was not aware of what was going on because he doesn’t follow up. So one of the activities we identified was monitoring, supervision and follow up by the Health Managers. The other one is sensitizing the District leadership on the need of having depression managed in HIV care system. We are saying the Health Managers and administrators like the CAO have to recognise the need for screening people with HIV for depression and managing them, because we believe that, many problems which eventually lead to death stem from that yet they would not have died. So sensitizing them on the need… and in the very first workshop we started sensitising them, because we had the Chairman there, CAO, DHO, they are already informed about the programme. We shall determine in future if there will be need to sensitize other mangers/administrators at the district hoping that if they are sensitized and understand the need, they will put in necessary effort in terms of resources; i.e. money, time or support to see that the programme moves forward. So building that capacity, and then strengthening the referral system – ideally we have a referral system. Some people said the referral system is weak – yet between the health workers and the community you cannot establish or strengthen the referral system if the managers are not concerned or they are not showing interest. So it is the health managers to strengthen the referral system by doing the needful. We shall look at the needful as we get into detail of the discussion. But so far we have summarised the activities in those three as were identified. There is a fourth one which is by mistake – orientation of implementing partner. In this map we have different shapes. Outcomes, activities and indicators were put/represented by different shapes as we shall see, as we move on. But orientation of implementing partners also featured many times, as we thought that these implementing partners, even if we are not the ones to train them, if they get to know, some can be with funds but not knowing where to allocate them. When they are informed about the purpose of this programme some of them may be supportive in different ways. The implementing partners can be some donor funded programmes (e.g. TASO) as you know those ones in the district. You told us that … one of the most important outcomes we can get at that level is buy-in and support. Outcome at the health organisational level …the district chairman, CAO, most especially the CAO – political buy-in, district administration most especially the top management, if they support the programme, that is very important compared to other things we may think about. If they look at it as if it is not important (Kizimuzimu), it will not move forward. And truthfully we have seen it in some districts and other areas that if the top management understands/appreciates the purpose/importance of a programme and support it, it moves forward and the impact is achieved. But if they do not appreciate the programme, you keep on trying but at the end you are struggling for nothing they will not play their role. If there is something to be done – that is not a priority. So if there is political buy-in and support from the administrators then the programme can be successful. They will allocate resources accordingly, they will prioritize as necessary. So we realised that one of the outcomes is increased buy-in and support for this kind of programme and then that will lead to allocation of more resources for managing depression in HIV. Allocation of resources i.e., finance, human resource, time and etc. so if they are in support of the programme they will allocate resources accordingly and once the resources are allocated, we can be assured that depression will be managed alongside HIV in our system, as we see in the map and once we have depression managed in HIV care, then we expect better clinical outcomes for HIV patients. If depression is managed along the way you find that other comorbid mental problems are managed. Such people will feel better and be more productive with reduced rate of medical illnesses and eventually consumption of drugs like coartem reduces because the key problem is dealt with as we shall see, as we move on with the discussion –that some of the health problems we neglect, when we address them there are several other issues you will have addressed (hit many birds with one stone). So if it goes like that it will lead to improved wellbeing of these people – and we want a healthy society. No leader would want to manage people with poor health/sick; they will not work, they will not pay tax, they will be crying, etc. So that is at the Health Management level.

When we get to the Health Facilities’ Level – at our hospitals where such work/activities as these are allocated, for what you suggested, Health Facility level is in the middle there. We identified activities like training the Health Workers in Mental Health Care, emphasising management of depression. Well this wasn’t suggested by you but these days we have new training package (*mhGAP*) mostly recommended by WHO. We have used it in some districts in Uganda. It tries to simplify for the general health workers who are not experts in managing people with mental Illness, to be able to detect and treat. So one of the activities is building capacity of the health workers but we shall not build the capacity of health workers to manage depression only because sometimes it may not be very easy to diagnose depression as we may think. We keep talking about feelings of low mood (okwenyamira) here. In the previous meeting, we talked/focussed so much on that because many people kept asking what depression is – when do you realise that you have depression if someone is just sad? So training – you cannot just train health workers in depression without considering other mental illnesses. There are mental disorders that can be comorbid. They will have features like those of depression yet it is something else or the person has both. So you will not manage one and leave out the other. I may be sad but not depressed. So building capacity of the health workers to be able to identify people with depression and manage them well. So one of the activities is that at Health Facility Level. Then we identified providing tools they can use to screen for probable depression. That is very important in building the capacity of health workers to be able to do a thorough clinical assessment and be able to understand that a patient meets the diagnosis of depression. Because one may come while depressed but their behaviour confusing/acted – because no patient will come and tell you that they have depression. So we need to put more effort on that. Also you mentioned that, it will be necessary to avail a start-up package of antidepressants because most health facilities usually have stock out. The antidepressants may not be readily available or when we sensitize and the programme picks up, we expect more patients who will need the medicine and the medicine may not be available. So if there is some start up package so that they can get the medicine, and when the district recognises the need for such medicine, they will make requests or purchase more medicine depending on the need. We identified mandatory screening and assessment for depression – you wanted that at the triage desk, but all the health workers involved in handling HIV patients; whether health workers at the clinic or community health workers coming to the clinic, like the VHTs who are sometimes at the clinics/hospitals, there should be particular time regularly for screening people with HIV to determine those with depression and those without. They might be depressed but not meeting the cut off for clinical depression like they only need some counselling because we said this is something broad – depression can be a result of a particular issue just for a period of time; so what is most important is that screening is done, these people are identified and then managed appropriately or referred to where they will be managed appropriately. Regular monitoring and support supervision at health facilities, then involving a psychiatric nurse in HIV care. It emerged that you have very few psychiatric nurses in the district and they are not involved in this work, yet we know because we shared that in some of the districts, the HIV clinics are managed by the psychiatric nurses for some reason. I think it is not the same in Mpigi or that is how it has been – please DHO you can note that as well. In some districts, psychiatric nurses are deliberately assigned to HIV clinics largely because they can do a lot of counselling. They can screen and because they also know that there are several mental health complications that come with HIV. So as you… “Did you say we have one psychiatric nurse in the whole district?”

E: HCIV has two.

F: But you can have many more than that. We have districts that recruit more psychiatric nurses because they can work as general nurses – they double actually. We have worked in some rural districts where the DHOs realise that they are much better and next round they recruit more. You deploy them, they can do any work anywhere but they can do what the general nurses cannot do. So the psychiatric nurses have to be involved in HIV care so that they can provide the expertise in identifying and handling the HIV patients. Those are some of the activities we identified at health facility level. Remember we said we do not need to identify 20 or 30 activities that may not be undertaken. We tried to be precise and consider what is doable and not just a wish list where we include everything – even what we cannot achieve. And when we get back to the map, I said these key components are represented by different shapes; the diamond represent activities, the rectangle represents outcomes. One of the outcomes we expect from the health facilities that is very important is, appreciation of mental health issues in HIV. That will of course be achieved after training them, building the capacity and so on – and this is very important outcome that once the health workers appreciate the mental health issues in HIV then it will be so helpful, because we have realised that people with HIV often get mental problems, and in the same way, people with mental health issues easily contract HIV because there are certain things they do unknowingly. They are more likely to acquire HIV. These two forms of illness are interrelated and if health workers are not aware of this, they will not prioritize this. It is true you said that health workers need to understand mental health issues in HIV care. When they get awareness, most likely they will be involved and the attitude will change. We realised that the appreciation will increase stigma and discrimination free services. Currently we recognised that there is stigma, we have some discrimination. Discrimination is broad. It can be done through action, through what people say, in what people do and the way they think, though thoughts usually lead to actions and what people say. The health worker can discriminate patients without mentioning it but by dodging to work in the HIV clinic or keep referring them to other health workers. So discrimination can be done in different ways. Stigma and discrimination was most common in HIV but it has reduced. It is still present in mental illness but we are trying to reduce it. When a patient has both HIV and mental illness, it is rare that they will not be stigmatized. So when the health workers appreciate the mental health issues, then hopefully the stigma will reduce. If stigma reduces, we believe that patients will receive required services and we shall get better clinical outcomes. Depression will be managed alongside HIV care and in the middle there some of the indicators (what will show that something is happening) we look at the number of patients seen and those who get better – remission, because we talked about the audit report. This is something we could do as part of the work – e.g. this programme has been running in Mpigi HCIV; for the past 6 months, how many patients have been screened and found to be with depression? Were they managed? How many are feeling better (remission)? If this is the number, after 6 more months, will you have the same number? But we expect that if the number of patients seen goes up, after some time we shall expect it to drop to show that the service is being delivered. But if it only continuously goes up, then it means there is a problem. That outcome will also eventually lead us to improved mental wellbeing of the patients.

The other outcome at the health facility level – health workers handling HIV patients can detect and manage depression once they have been trained and there is increased appreciation that again leads to better clinical outcomes. The assumptions we discussed previously at facility level, e.g. health workers getting involved, we assume that health workers are available and will be knowledgeable once trained and they will be available. These assumptions are mostly at administrative level and also at facility level, administration has a hand in them because part of what we assume, administration has the authority to make adjustments e.g., if we talk of health workers being available, they may be trained but administration gives them transfer to another facility. E.g. Mpigi HCIV which is involved in this programme, health workers can be transferred to Buwama and we shall have health workers with limited knowledge and we may not be able to train all health workers. The other assumption in the middle there is Health Unit Management Committees (HUMC) are active, because they have a role to play Mr. DHE; but are they active? So if the HUMC are not active, some of these things are not likely to be realised because they have to do some follow up. So if they know and are actively involved, they will follow up. But we are assuming that they are there and will be active – ‘in one of the health units there was a complaint of stock out but when the store was checked by one of the psychiatrists, the medicine was available and discovered it was intentionally not being dispensed to patients in order for them to buy the medicines at the drug shop’. So those are some of the challenges we have in our health system. So sometimes we talk about challenges but have not gone in detail to understand the cause.

There is another assumption that medicines and logistical supplies are in place. We have talked about capacity building, the health workers are active and involved, they are handling, they are detecting and managing and once that is happening, we assume depression will be managed and we shall have clinical outcomes. But one important assumption there, is that the medicines and other supplies are in place because if there is no medicine, the capacity is built but the health workers cannot deliver. So some of these assumptions, the administration has an upper hand in them because they have to make sure drugs are available. Some districts have performance based funding (I do not know if Mpigi has it) and you can choose how to use that money depending on your local needs. So we do not have to depend on only National Medical Stores (NMS). We have seen that happening in some facilities – in some districts there are health facilities that continuously complain about stock out while others do not complain at all because the in-charge endeavours that with all funding received, drugs are available. Those are assumptions – to take us back briefly to health organisational level, one of the assumptions is that we have political buy-in and support because the CAO coming here to participate doesn’t necessarily mean that he will be supportive. So that is an assumption. Much as the chairman came for the launch you can’t tell if he will be supportive or not but we assume these people will be supportive.

The other assumption is flexibility in medicines budgetary allocations. Whereas we complain about stock outs, every district has that provision, that is, why we say NMS has its own challenges as well. Available flexibility is that more antidepressants can be requested for if needed and less antipsychotics if less needed. Though the empty/used up tins needed to be first presented, but currently a request can be made for drugs according to demand. That is the provision that NMS has. It is not a matter of increasing supply of all drugs for every facility to have drugs in excess, but we can make an exchange/ substitution. So that is an assumption. The other assumption is supportive policies in place and we hope the administration will be supportive and an efficient HMIS system with the biostatistician active and involved. I put more emphasis on this because there is data collection besides this being a research project. When it comes to planning, it will be difficult for the DHO to defend her argument before the council, CAO and the chairman, that we need this if it is not backed by data. If The HMIS system is weak, then you cannot verify that we have this number of patients and this needs to be done. We are assuming that Mpigi HMIS system is active and has right information.

The other key level is the community and in those two workshops we learnt that a lot of HIV management is getting pushed to the community. We called it the Community Led ARV Distribution programme (CLAD), so we are shifting more to the community. For those who were part of the previous meetings, out of your views, the activities we identified include; sensitization of PLWHA groups or the expert clients on depression in HIV care. Expert clients do a lot of work here. We have the multiplier effect as well – because they understand HIV patients more than health workers at the facility, so they are more helpful. We have training health workers to screen for depression in the community, create demand through community sensitization – if the DHO is at a talk show, community radio and a few of you who might be involved can sensitize people and create need as they get awareness. In the community, the other activity was to empower people especially those with HIV to fight poverty because poverty is also one of the causes of depression. If people can be empowered to deal with poverty; like before TASO was extending a hand to PLWHA by giving them food and other items. ‘Patients indeed need support’. Then monitoring and follow up in the community – it was, however, not clear on who should do the monitoring and supervision in the community but the point was captured that we need to follow up on activities carried out by VHTs and expert clients but most especially patients who have been identified and are on treatment.( someone had been on ARVs for long but had a breakdown due to depression – currently how is this person?) and we need to be realistic because the health workers will not leave hospitals to come and monitor patients in the community, so who is responsible for that? Whether expert clients, we shall discuss this further as we move on. Also integrating and emphasising this issue during the health talks. Some of us get opportunities as VHTs, expert clients, health workers etc. to conduct health talks – can we talk about integration of depression in HIV care even if it is only for two minutes as long as the community can get it. There are some more we shall be adding. We realise that in the community, if activities are done as supposed to be, we expect to have increased awareness in the community and once we have increased awareness PLWHA will be empowered thus seek help. So we expect increased help seeking for HIV patients having depression. Similarly there will be increased demand for services. Sometimes the services are not available but when there is demand or the need is there, but demand is not reflected. We are aware that there is need so we need to increase demand such that people can seek help. Once we realise that then we expect to have more patients with HIV receiving treatment for depression as an outcome. All others we talked about were short term outcomes. This could be an intermediate or long term outcome – more patients receiving treatment and when we have more of them receiving treatment that will lead to reduced incidence and prevalence of depression among HIV patients. Then, in presence of this we hope to reduce the rate of depression among HIV patients. They will be receiving treatment and getting better and those who are not yet depressed, through the health talks, they will be able to learn what brings about depression to prevent it, meaning reduced prevalence and incidence. So we shall be expecting the number to reduce and we get very few new patients and subsequently this should lead to better clinical outcomes – there will be recovery and therefore better clinical outcomes. One of the indicators is increase in disclosure among PLWHA. Some of the assumptions we made there include; effective HIV care system. We are yet to establish how effective our HIV care system is even when CLAD takes on because in some districts, they have some challenges – unless a patient goes for refill, interaction between the health worker and patients is limited. The expert clients are sometimes not available or probably they do not know what to do. When a patient is tested and enrolled on ART, what is considered important is, a patient recognising that they need refill and adhere to appointment date without assessment of how they are/ considering their condition. But we are assuming we shall have an effective HIV care system. The details of what an effective HIV care system entails – we shall talk about them during the discussion. Another assumption here is that there is an efficient referral system – that people know when and where to refer. We began by saying that the administration should strengthen the referral system and it is coming back also in the community. So we are assuming that the referral system is there and efficient. And when we talk of referral system, there has to be ‘back referral’. It could be at HC III and the patient has HIV, is very depressed and suicidal. We should know where to refer them than subject them to risk of death. The other assumption is; facilitation of expert clients (I’m not sure if expert clients are facilitated, hoping that we shall not assume that MRC will always facilitate the expert clients) and then the routine follow up in the community – we assume that should be part of an efficient HIV care system. So those are some of the assumptions. So in short, that is what we have so far extracted and summarised from the two discussions we had. Like I told you some points were repeated, some with same idea and we did not have to write everything. You can come up with 60 points but end up with 10 because some of them have same ideas. A good programme does not have to be with very many activities. So currently that is where we are. We all have copies and as I said as we get into the discussion we shall want to review – first of all to confirm that these are our ideas from those two workshops and maybe we identify areas where we need to add, adjust and eventually we refine and get a better map – slightly better than what we have so far. Are we together?

Now the next part – we still have some time to reason together. Each one of us has a copy and the discussion is now open. So far this is what we have, how do we/what is needed to refine it? But to be precise, let us begin from the first level; are these the activities required at the Administrative level? Do we need to add more or deduct some? What needs to be done? When we talk about Administrative level, we are referring to managers, CAO, the Secretary Health, DHO etc. So is there any amendments we can make on that or is there any other outcome we expect?

And may be, one important one we did not want to be dictators, we wanted to first bring this back to you for discussion so that we can see how it can be refined – that is the “impact”. We still have three impacts but when we are making the map, we do not say impacts we rather say an impact. So out of the three impacts, which ones can we say that this is an outcome so that it can be shifted backwards? So we should start from there before going to another level. We have; management of depression integrated into HIV care as one of the impacts, we have improved mental wellbeing among HIV patients – does it qualify to be an impact? We have reduced mortality attributable to depression in HIV care. I have said these are your ideas we haven’t added ours, but when we are refining we shall modify accordingly. So we are still discussing your ideas that is where we are. What we called reduced mortality attributable to HIV means reducing the number of deaths related to depression in HIV. So what are the two impacts that we can move backwards to become outcomes or which among the three should we take as our impact? You can rise up your hand and give in your contribution.

R: I think reduced mortality attributable to depression in HIV should be the impact. After putting the management systems and people being well our impact will be reduced mortality therefore I take it as the impact.

F: That is what you think?

R: Yes that is what I think, then it can be followed by improved mental wellbeing and management of depression integrated into HIV care.

F: Since it is a discussion let me ask you a question; if we take it as our impact, currently do we know the number of people who die due to depression in HIV? Can we say, we have many people dying due to depression in HIV?

R: The death is not attributed to a single cause it is a combination of factors such as depression, not getting treatment so they combine ultimately leading to death.

E: I wanted to contribute; in mental health, we do not usually look at mortality because for example suicide – the rate of suicide in the population is about 7 per 100,000. In mental health, we tend to look at more of the mental wellbeing – the way the mental health is affecting the persons functioning because mortality is a very small part. Most mental illnesses will not lead directly to mortality. It is not like TB, when you get TB and you do not treat it, somebody will die by the end of the year. Mental illness does not usually do that except for only suicide, because when we confine ourselves to only mortality then we miss out a bigger part of mental wellbeing.

F: Ok, before we proceed have we understood that point?

R: Yes.

F: Sister is that related to this point or your rising another point, ok you are rising another point, may be let us begin with DHE.

R: Me, I would suggest that we first integrate then these other two indicators will come as we continue with the process. Let us first integrate depression into HIV then after the others will obviously come along.

F: It is obvious that once depression is integrated into HIV the others will come along.

Yes, is your comment related to this?

R: Me I think if we integrate, we shall not have people dying... people getting mental…will not be there because we will have managed them.

F: We were in the second group where this came as an impact but I think we are now reaching an agreement that reduced mortality attributable to depression in HIV is going to be removed from the impacts, because we do not know the numbers of death as a result of depression in HIV and you also commented that it is not depression alone but many factors lead to these death. So we have agreed that we shall put it in outcomes in a rephrased form, but at the end of it all whatever impact we choose here, once it is realized even this will be realized.

F: Now we are remaining with two, that is to say; management of depression integrated into HIV care and improved mental wellbeing among HIV patients. Which one among the two can we take as the impact of all that we have done?

R: For me, I want to remove management of depression integrated into HIV care because we can integrate depression management into HIV care but at the end our aim is to have improved mental wellbeing among HIV patients. So I think it can also be shifted to outcomes.

F: Thank you so much, have we all understood him? Sister have you heard his point because you were not attentive.

F: May be for emphasis of his point, we are looking at these two; management of depression integrated into HIV care and improved mental wellbeing among HIV patients. Our friend here is saying that management of depression can be integrated into HIV care but we may fail to have an impact if people are not committed to their work, so he is suggesting improved mental wellbeing among HIV patients to be our impact.

F: What do others suggest?

R: Mr. Facilitator, for me I was suggesting that the impact may not be one. We may remain with the three impacts as they are or we can make additions.

F: Do you have one that you can add?

R: Yes, I have.

F: Please raise it up.

R: As I am the focal person for gender, I have been listening attentively as you were presenting. There is where you said that it does not make sense for a doctor to present a budget and it is rejected but as we are preparing this, I have not seen how the gender component is being addressed. So that may make our budget to be rejected because it is not gender sensitive. Remember when you are mainstreaming gender, it begins from planning up to budgeting and once you miss one step it ceases to be a mainstream. There is where you mentioned about training the health workers and I think during that training, we should have a component of integrating gender in management of HIV and depression targeting the health workers but we are also going to target the beneficiaries – the clients because they have to be aware. At the level of clients we shall be telling them what gender is, what is the role of gender in management of depression? Then at the district level we shall train them in planning because we need to come up with a gender sensitive budget. So I am just proposing that at the end our impact will be “A gender sensitive community.”

F: So you are saying the impact will be a gender sensitive…

R: A gender sensitive community or a gender sensitive budget.

F: It cannot be a budget.

R: Ok, the impact can be a gender sensitive community and then there is also another one,

F: Or we first finish the one of gender, is it also on gender?

R: Yeah, it is on gender because I was trying to come up with activities, then assumptions and then the impacts. Ok, from impact, assumptions and then the activities or the interventions.

J: Sorry I have flu my voice may not be clear. But when we are looking at the impact in the Theory of Change we look at it in two forms one is the process and then the other one.

Now there are these first levels, orientation of implementation partners, appreciation, increased awareness, increased demand, all those involve the process because all these are interventions and we are trying to impact on the system. Now for your concern to be taken in consideration we mainstream gender into all these. For example, when we say increased awareness it involves men and women, when we say increased demand, how many women and men are demanding. At the end when we say there is full recovery, we are able to know how many women and men benefited from that programme and then also on improved mental wellbeing, it can be disaggregated into the two genders (male and female). So in that case gender becomes a cross-cutting process/ issue so we cannot create its own impact entirely.

F: Thank you, I do not know whether sister you have understood him clearly?

R: I have understood him well. It is actually good but there is where you mentioned health workers assessing a person who has HIV but sometimes there could be other factors that could have caused that.

F: Ok, you said you are the gender focal person, I get your argument very well, I understand the component that you are trying to bring out. Just like he has said any component we are talking about concerns men and women. Because when we talk about gender it does not discriminate men or women because some think that gender issues are for women alone yet that is not the case. One time we were writing a proposal with World Vision but for them they did not want to use the word ‘people’, for World Vision you had to use the words women, men, boys and girls. I first refused because we were a small group and I argued that but really if we say people what is the difference? Until when someone said the donor is World Vision and if that is what they want, that is what you should include in the proposal so we went with that. But when you talk of women, men, boys and girls you are talking of people. Now here we are talking of men and women. Just like he has said we cannot say we are having an impact specifically for gender, the best we can is to mainstream gender at every point since it is a cross cutting issue. In any case your point is good because we need people to appreciate the gender issues in that there could be problems that specifically affect HIV infected women or men, so such issues will feature not only in the impacts but everywhere we have outcomes or interventions. So that is what we have to look at; be it capacity building, training or orientation, how does gender feature there? I hope we are together.

J: So she can help us to check whether gender is represented in whatever we are doing in integration. Every activity from this programme is it gender sensitive? When we are collecting information, when doing trainings or any activity. So that means even the impact that we shall get will be gender sensitive.

R: When I look at this it is good if we have section where the gender activities appear.

J: Now we are going to get to another level, because this diagram up to now, it is still very congested and even when we get to office, I will still have to discuss with him because it is very congested. If we continue to disaggregate it, it might fit like on six pages.

But when we shall be building interventions and monitoring tools, say like for example, increased awareness will be disintegrated among say, for example, if it had children we would say among children ,women and men, increased demand for services among children, men and women, empowerment of PLWHA, how many were women and how many were men. Every activity here will be given a gender flavour such that the outcome there also has a gender flavour, the major impact will also have a gender flavour – that is to say improved mental wellbeing among HIV patients, how many will be men and how many will be women.

R: Actually, may be, my last point is “we normally talk about numbers that may be, we are going to target seven females and six males and as such, we become satisfied that we have covered that area of gender but, in our annex we have to explain what are the issues of those women, what are the issues of those men and how have you tried to address them. We have to explain it because it is not about the number of people, the number of women or men if we just say it, we shall have not identified the issues and addressed them.

J: Let me further explain it, down on the Theory of Change we shall draw a line here, just because he has not yet drawn it and we shall call it context. It is for that reason that I was hired to look at the context or quality of things – what is called qualitative. Even Christine and the gentleman next you, we shall be looking at the context so that we can add value on the numbers. So that should not bother you a lot because it is the main reason for my being in the project. So down here, we shall draw a line, I think you are actually reminding the facilitator to define the context? Such that if it is many women who have not taken up the services why? If they have not benefited why? If it is the men who are more empowered why? So that we are able to respond to what they call ‘Emerging and Re-emerging needs in the implementation context.

F: I hope sister you are comfortable now. The most important issue is gender sensitivity. The map here is summarizing issues. We cannot unpack everything on the map, but when we unpack, these issues will come up especially in the write ups that we shall make but the map is just a summary.

J: Joshua there is another issue that this gentleman has raised and it is very important and I liked it, if we say management of depression integrated into HIV care, actually, I am just adding onto what you said; Museveni says, what is your task? So our task is management of depression integrated into HIV care, which means it is not the impact. That means we cannot take it as the impact. So that means the whole of this diagram is addressing that so we have to rethink, and also professor said that mortality is not common in mental illness but he earlier on told us that we shall be here for 18 months but understanding mortality and nutrition you need a lot of time. For example, saying that this child is stunted you need to study that for a long time. Therefore if we stick to mortality we may not achieve the main objective as to why we are here yet there are other things or parameters that can be measured over a period of 18 months to show that our objective has been achieved, just like the other lady who is qualitative asked what are those things that we can be able to see that this has been achieved.

F: Thank you so much, so those ideas further support retaining improved mental wellbeing among HIV patients as our impact. So do we want retain that as our impact for this programme? In that when we integrate management of depression into HIV care we eventually get improved mental wellbeing among HIV patients or do we want to add more impacts or do we want to change the impact? What is our suggestion?

We are glad to have you with us deputy CAO, some of us did not know that you are around. In most districts, deputy CAO is always in charge of health directly. So we have been talking about things such as buy-in, support etc. but let us hope since the deputy CAO is with us all that will be considered. So are we agreeing that we retain improved mental wellbeing among HIV patients as the impact of this exercise?

R: Yes.

F: Yeah, because it is consensus in the first place and we are not doing an exam, therefore we do not have a right or wrong answer, therefore whatever we think is right is what we shall adopt for the programme. Ok, so we are going to adjust and push back these two (*referring to ToC map*). So, let us also scrutinize the outcomes and interventions, assumptions and… Yes sir,

R: I was commenting on interventions,

F: No problem.

R: Interventions you talked of district levels 1, 2, 3, buy-in, orienting implementing partners,

F: Yeah, we took it into interventions.

R: Increased buy-in and support should not only be at the district level we should also engage the people at Sub County level.

F: Ok, the right word should have been Health Organization level, may be, or Management.

R: Yeah, but you were talking about the politicians,

F: What I am trying to say is that, we have administration or management at health facility and you are saying we put Sub County and then another person will tell us to add Counties assuming they are active.

R: Counties are not active, but on buy-in you talked about politicians.

F: Yes.

R: Even at the sub county they are there, it is a government.

F: Yes, local government.

E: Or we call them policy makers and implementers.

R: Because they are closer to the health Centre III s and to the communities, so it would have been district level and sub county level.

F: Ok, so what I am saying we are just changing the word from district level to health organization/ management level.

E: What about even taking it higher? What about at the national level because even at the ministry you may find they need buy-in at that level. Why do not we call them policy makers and implementers?

R: Or indicating the levels?

F: No, of course we do not need to indicate the levels here in the map.

R: It was misleading putting there the word district.

F: Ok the word district is the one we are going to change, thank you so much. Yes we are scrutinizing interventions and outcomes to see what we can add or remove, something.

R: I was suggesting that the district level would remain there then just like your colleague said you are going to explain what people you will be targeting. For me I see that it flows very well that from the district level you have certain people you are going to target not all but you have specific people then you come at health facility, still you have specific people not all and even at the community not all because you will target the beneficiaries.

F: Ok, to make it clearer, we are talking about structure not infrastructure. Here, we are talking about services – in service delivery we know what happens at the community, the health facilities and the administration level. Under administration, we have district. In the future, districts may be replaced with provinces or regional tier yet in our write ups we have district. So, that is why we are suggesting to have a uniform word talking about administration level or Health Organization and Administration. Because if the word district is changed in the future, our write ups may become confusing. So the word district will be removed… because he (*referring to the previous speaker*) was right, even a sub county chief also has a role to play, so if you say district, he will feel left out.

J: Or she has a suggestion?

F: Ok.

J: What can we use?

E: What about policy makers?

M: Yeah, the district, I wanted to explain that when we say district we are limiting ourselves to district and anything within district and yet we know that above the district level, we have the ministry, the commissioners and the minister for health. So if we begin here and stop here as a sealing, then we are cutting out actually the most important because all the budgets come from above. So to make it broader, including the top managers – I think health management and policy makers would help including the district

F: But not health policy makers, we are talking of levels like we said community level, health facility level, now the word should be talking of that level. is it health organization and management level or …

M: Health management.

F: But not health managers.

M: Health management.

F: Health management and organization because that is now broad or manager depending on the local administration.

R: The nomenclature above, is binding all of us.

F: Yes, it brings us all together. We are continuing with the discussion we still have time.

E: So are we still at interventions now?

F: We are looking at both interventions and outcomes now or we could even look at the link but I am not limiting you. You can even say some of the assumptions we are making, may be are not possible in Mpigi because you understand your district better.

R: Ok, I am taking you back,

F: No problem,

R: You said previously that there has been less knowledge as far as mental conditions are concerned especially among the health workers. Now looking at our impact, which is improved mental wellbeing, I make an assumption that due to lack of capacity to diagnose mental conditions before starting the programme, and then improving this capacity through training since it is among the interventions, there will be more of the mental conditions being diagnosed which may confuse us on whether we have achieved our impact of having improved mental wellbeing among HIV patients? What I do not know is that, do we currently have the figures regarding improved mental health in HIV patients?

F: Have we understood his point?

E: I thought you mentioned something like that, you said that at the beginning we may see the cases going up but if the management is happening eventually they will reduce, in his talk he mentioned it.

F: To further emphasize, remember when we talk about common mental disorders in most cases we are talking about depression and anxiety and the two are inseparable. Some health workers get challenges in separating this but when depression is managed even the anxiety is also managed. So if the programme is handling depression well even other mental health issues associated with depression will be wiped out so we expect people’s mental wellbeing to be fine. We also said that we expect the numbers to go up initially and then come down that is when we review the HMIS so that will show us that people are now well.

We all agreed that depression among HIV patients is there but it is not diagnosed or treated in most people. So we hope that, any programme that will integrate depression management will begin with sensitization, screening, diagnosing and treating depressed clients. Therefore we expect the numbers to rise if the programme is run actively, because HIV clinics will report more numbers than before but these will eventually reduce because most will be treated and even with mental health promotion through sensitization, the number of new cases will reduce. Are we together so far?

E: Capture the issue of health promotion.

F: Health promotion,

E: Community health…

F: Because when we talk of improved mental wellbeing there should be health promotion. Health promotion, can we bring it out as our outcome or intervention because it is implied in our impact but maybe we need to state it somewhere?

M: Could it be embedded within better clinical outcomes for HIV patients?

E: But promotion even happens…like we saw that in domestic violence when you reduce on violence, then you have contributed towards reducing the incidence.

F: Our colleague is reminding us that health promotion is generally very broad “gigantic” and many of the interventions here or outcomes in one way or another are linked to health promotion. Because in the sensitization we tell them the risk factors, the causes, the preventive factors or protective factors because if the sensitization is done promotion has to feature, we have to tell people about the causes, about things they can do to avoid these problems.

J: Where does the point for linkages and other community programmes come from?

F: Income generating activities, was it in assumptions? Ok, empowerment to address poverty that is now an intervention. Yeah, it came under interventions.

J: Is it number 12? (*Referring to the ToC map*)

F: It is an intervention number 13. (*Referring to the ToC map*)

J: Ok.

F: We still have it at intervention level.

J: I have seen it.

F: Any more contributions? I have not heard any voice from this side, yes my brother any question?

E: Is the community only screening, I mean expert clients because we are going to bring there a programme which says the first level will be offered by a health worker so may be an expert client.

F: Because we expect the expert client especially to be offering this treatment. That is what we had said that there is point 16 intervention that is not on the map that we are going to add, that is, training expert clients in offering brief psychotherapy in the community.

E: What do you think on the treatment…?

F: At the health facility? But shall we be able to keep… if CLAD is coming on board? Since we said that one person will be representing the group, then we hope that community health worker or expert client should be able to help themselves at first because even detection of health problems is so much at the community, that is why the VHTs and expert have to be involved. We are not talking about screening only but also offering the initial help before referring. That is why we are adding an intervention there “training expert clients to be able to offer brief psychotherapy”.

E: Can we hear from her?

R: It is necessary.

R: Yeah, it is what has been happening, ideally those people have trainings in the other aspects of HIV but now what is needed is to add them knowledge in the aspects of mental health and it is among the roles that currently they are doing.

F: So we have already added that as one of the interventions. Yes…

R: Yes, I think there will be a referral system whereby, if they have improved you send them to the VHTs to continue with the treatment.

F: You refer to the VHTs to continue with the treatment?

R: Yes.

F: Which treatment?

R: Of depression.

F: Which treatment in particular?

R: To give to clients the treatment of depression.

F: Which treatment? Ok, because treatment of depression can be psychotherapy, counselling sort of or medication.

R: Both.

F: But the VHTs are not offering medication for depression, we do not have community health workers giving out the antidepressants. So, are you saying they refer to you, you give the medication and then you refer back to the VHT for psychotherapy?

E: Follow up, follow up.

F: And follow up.

R: With counselling

E: Actually this project believes that actually the health worker can then refer this person to the…

F: Yes.

R: Now in the community, CLAD also has steps or guidelines. In some instances a person may not qualify to get treatment from the community and as such they have to come to the facility. So in brief ,we cannot shift all the interventions for depression to CLAD because, like for example, if a person has poor adherence to treatment, they are referred to the health facility for better assessment so in case there is depression this person can be assessed at the facility.

F: Let me hope you have just not forgotten because when you look at intervention number 7, the CLAD issue just came in because the shift is coming, otherwise all the management has been at the facility level. Number 7 is talking of screening at the facility – screening for depression at the health facility.

E: Also in the model if you attended the presentation… how you can manage depression at different level, there is where it is called mild depression, moderate to severe, severe and when somebody is suicidal. Those very high levels have to be managed at the health facilities or even beyond to the regional hospitals or Butabika National Referral Hospital. So there are levels even on depression depending on the scores somebody gets, mild forms which can be managed in the community, then severe forms which can be managed at the health centre then there are some which we have to refer to the highest level like Butabika or may be a regional referral hospital

R: I may not be around but I am proposing that we should not only talk about demand, but we should also talk about the way these people can handle patients who are depressed. You know sometimes people come from health units and when they return to their communities, their conditions are worsened by their communities. During this sensitization – creating awareness, let us not talk about demand in such a way that we only call for numbers of people, but we should also teach them how to effectively handle the patients seen. I have just brought that as a method of work during the implementation of the proposed activity. Then regarding self-help/ empowerment to address poverty, I know you are going to engage these people to embark on income generating activities but emphasis should be on how they are going to share this because you may find that in a house hold both the husband and wife are partners in the project granted to them but when it comes, for access it is ok, because they can both access that project but when it comes to ownership, it has been evident on many occasions that women get so much depressed although there are rare cases for men, but women are so much depressed as a result of being a victim of other depressive issues and in addition be affected by the project that she has been given as she is denied control over the Project and when it comes for benefiting from that project , it is the man. Men have tactics that they apply so that women do not benefit from the proceeds of the Project and this makes them more depressed (depressed twice). During the Proposal writing, the above concerns raised should be considered and included so that it is explicit in the document how these women are going to benefit and how the men are going to benefit from those Projects.

F: Thank you the gender focal person, your point has been taken.

E: May be to add a point as James said, we cannot play all these roles, like empowerment, I mean addressing issues of poverty that is really outside our rim. We shall interact with other agencies which are specialized in that area, we shall just say this person will benefit and we refer them to a resource which can address poverty but we will not address poverty in this project.

M: Just to take you a little bit, some question was asked whether it was ok to train some VHTs and expert clients on the basic counselling for people with depression, then someone said yes it is important. But I think we also needed to hear from some representatives here whether they are happy to take it on themselves because I remember in one of the ToC workshops, a point was raised that some HIV patients are not very free with either the expert clients or the VHTs.

F: With the VHTs.

J: One of the group. So we want to hear from you first of all are you happy to do the counselling and do you think when you do it, it will be impactful because there is no use doing it when someone you are talking to is not really trusting your advice.

E: Actually we are going to have a workshop which is going to be scheduled where we are going to have a presentation of the psychotherapy and we shall probably discuss that in greater detail because the whole workshop is going to be about that.

F: We discuss who will be targeted for…

E: And whether people are comfortable we shall actually bring the same group.

F: And Mr. DHE before you came in, you were not in the other group but it emerged that the community health workers are not trusted by the patients, mostly they prefer dealing with expert clients.

R: I wanted the issue of strengthening referral to come out clearly because if I look at this one – increased self-awareness of people living with HIV, it cannot come out clearly if the referral system is not well arranged. So in the community there must be a tenuous referral system that can enable to enhance this health indicator.

F: Now we talked of self-disclosure, this is coming as an indicator here. Strengthening referral already featured as one of very first activities although it was at the management level, because this referral is not between the CAO and DHO or at your level but you are the ones to strengthen this referral. Right from the bottom we have health centre I, II, III, IV We already have strengthening referral as an intervention.

J: They are trying to clarify what is really important in the context, it might not be that they are bringing new ideas but they are trying to elaborate how the context is likely to play.

F: Are you talking of referral within the community?

E: We need cases to come from the community, those who are doing well.

R: When the expert clients and VHTs are well informed of the services that are available, it can increase the referral system – people can easily be identified and then referred for management.

F: Emphasizing referral, basically which we captured.

E: Is it possible to put number 1 at all the levels?

F: Because how can we say one intervention in the community is strengthening referral and then at the health facility strengthening referral?

E: Ok, strengthening is an activity of the managers.

F: And then because we are sensitizing the community, the health workers and then we are strengthening referral at all levels – actually referral and back referrals, in fact because there should be back referral as well.

J: His argument is that if we are focusing on strengthening referral we need to go beyond institutions.

F: We captured that. Yes madam,

R: Actually people in the community are not aware that depression is an illness but as we go along sensitizing and health educating the expert clients and the VHTs about the signs and symptoms, they will be able to make appropriate referrals to the health facilities. But the community does not know that depression can cause death, but when it was tried out last time, many people were found to have depression and on treatment they recovered. Just like he said the graph will first rise but will eventually decline. When we start implementing the programme we shall get results, those with depression will be identified, managed and will recover.

F: When you look at intervention number 12 and 15 all are about sensitization, community sensitization, emphasizing this during the health talks.

R: Training them.

F: That one we added, it is number 16. But sensitization, we are informing the community about the existence of the problem. That is what number 12 is talking about.

R: In cases of community outreaches, antidepressants can be packed along with ARVs such that for a patient with suppressed viral load but with depression can be managed in the community.

F: So during training, that will be streamlined because mental health drugs may not easily be dispensed by everyone, say VHTs, because sometimes assessments are needed and dosages may change.

E: In our project, we do not only emphasize antidepressants only but we want also to offer psychotherapy – brief psychotherapy, so that you are able to talk to a client well in order to influence his /her thoughts about depression. So we want those things. We think the Expert clients or community health workers may be able to do counselling as well.It was implemented in India and found that they could offer counselling at a very high standard, if they are well trained. So we want to see whether we can also use our expert clients here to provide that brief counselling.

F: Yes, madam.

R: As they are doing that counselling, of course they have to be guided in terms of where to receive services. I do not know whether you will have provision for referral forms and teach them how to write and refer patients to health units.

F: Currently, how are patients referred?

R: I am just asking, I do not know whether the system is in place, but it would be good practice for record purposes.

F: Are there referral books.

R: They are available.

F: They have the referral books especially in the community used by VHTs,

R: You have talked about the referral books, is it the referred patient who goes along with the book or he/she has a form that he/she presents or he/she just presents him/herself verbally? .

R: The one who refers the client is the one who fills it in,

F: Ok,

R: Takes it to the health facility. (*Referring to the client taking the form to the health facility*)

F: Ok,

R: After he/she has been reviewed, the referral form is filled in and signed by the reviewer as well as the client, as an indicator that he/she has been reviewed at the health facility.

E: Referring this patient because I think…

R: Since it is a study, from experience of the previous studies, we have been observing that sometimes there is need for documentation of data which is required so much,

F: Yes,

R: But, in reference to the health systems and as I have been hearing as well as I know, many times workload is the ‘common song’ (*probably meant* *complaint*) in health facilities. I have no hope that Mpigi will be any different from other districts. We have talked about training of the available health workers, but still, I think there is something missing that should have been included in this project as it would facilitate improved documentation, then; management of generally mental health as I know it, it requires some extra time,

F: Yes,

R: In terms of diagnosis, and now in addition, there is documentation. I would have been very grateful if that kind of thing appeared somewhere around here.

F: Which kind of thing? Intervention, be precise.

R: Intervention, that kind of intervention, maybe, if the program could recruit, maybe, data clerks or some kind of human resource to boost capturing of data.

F: Now, programmes sometimes are tempted to include things (*activities*) that are not sustainable, for example if whenever we implement a project, we recruit someone to be capturing that information only/recording, when the project ends, his/her service also ends and what follows next is loss of information, that is why you see that most programmes prefer working with the existing staff and structure for performance improvement. That is the reason why, one of the assumptions we are having, is an efficient HMIS system. There is HMIS system which is broad to facility level where reports are submitted, but we are hopeful that HIV clinic, is keen at keeping records and data, isn’t it?

R: In study situations, it is usually different because you may find yourselves in need of data for,

F: Data for patients.

R: Because I have already heard you talk about …, Remember, I was saying that, even the assessment of mental health itself, takes a lot of time, you may find one client (*of mental illness*) maybe, takes a lot of time compared to those without mental health condition and so, I am saying that there will be challenges of,

F: In what? In keeping data.

R: Of over load,

F: Screening,

R: And in the end it will be found in documentation.

E: Ok, there are two things, one thing is we don’t want to set up something which is not sustainable. We are going to do as minimal intervention. Maybe the only thing we are going to do is to strengthen the expert client – is the only additional thing we are going to put in, but we want the health systems to run almost as it runs regularly, and that is why we have even confined ourselves to depression management, and depression management, we expect you to use – we are going to use the screening tools so that we do not overload you with having to assess the psychosis, assess cognitive functioning, no; just some simple questions to assess for depression. Few questions , I mean even like the initial screening, is just two questions, the PHQ2 , just 2 questions screening for depression so that we don’t over load. We know the health worker is already over loaded with things. But now for purposes of our study, in order for us to be able to say that this thing works or not, we are going to hire research staff, who will collect data and when the study is over, the research staff will go away but we want the system to work as minimally uninterrupted as possible so that our main input is going to be putting in the training, maybe providing the IEC materials, skills and all that. But we do not want we want a system we want to make a system that will run when we are away. We do not want to really put in things which are not sustainable. We want to use the drugs that we are using, the antidepressants that we are using, the staff who are available, the expert clients who are there, maybe give them some more skills and maybe give them some little responsibility like to undertake brief counselling. But the research part is going to be a separate system that we are going to put in and pull out once the study is done and it will not be providing care, no, it will just be monitoring, capturing the information….

F: Was I going that side or this side?

R: There are NGOs where we refer our clients when they develop a concern like this one under discussion (*depression*). An example of such NGO is Kisomu,

F: Kisomu?

R: At Kisomu, we have to refer people like those who have orphans as this issue can be a cause of depression among people,

R: But now, if such organization is not represented here, we are going to meet hardships as we refer patients to them due to knowledge deficit of the project and its objectives.

R: Why do you normally refer them? (*What is the purpose for referral/what do you want them to benefit?)*

R: They are given books, school fees paid. We refer them and are given books, school fees paid for while others are given food where need arises.

F: He is talking of some few existing agencies where they refer some HIV patients for material support like he is mentioning, school fees, exercise books. So, he is saying, they will find it difficult when some patients are referred to these agencies and yet the agencies are not aware of these programmes. And I wanted to react immediately that no, this program is strictly talking of HIV – managing depression among these patients (*probably meant depression management among patients with HIV)*. You’ve already been sending them there, you are sending them for books, for school fees, not for mental health issues, yes, so when you send those who have been identified with depression and have been treated, I do not see any way they will be rejected because there is some other program. This program is not doing any material support, are you… oh, sorry! You have been referring them there, you have been referring them there when they are sick and also to get some assistance when the prevailing circumstances are harsh. Now, this programme focuses on treating you and so, when you refer patients to the other agencies, these agencies will not need to know whether our programme treats you or not. If the agencies have been offering books, there is no way they will deny the one with depression, the opportunity due to knowledge deficit of the programme. I do not see how this can frustrate the other. However, we shall emphasize that if those patients do not know where they can be referred following depression as a result of poverty, harsh environment, the expert client shall connect such patients to agencies from where they can get appropriate assistance e.g. Kisomu - Connecting the patient where he/she can access assistance, amelioration of his condition, and maybe, attain improvement. We have said that this is going to be emphasized at community level and it is not going to frustrate treatment of community members.

E: Also another point is that; what he is also saying, orienting the implementing partners or other partners maybe, may create awareness in such partners like that one, about depression, yes, even those people who are providing the material support, maybe we can talk to some of those agencies to tell them that there is also an issue of depression such that in case they see such a thing in one of their beneficiaries…

F: Which they are providing and who is going to, to raise the awareness?

E: I think the district is going to,

F: Ok, implementing partners,

E: And maybe, as long as you have put it.

F: The external agencies. Yes, it is good the DHO is here,

F: We are talking of sensitizing or any other partner who are involved in HIV care directly or indirectly maybe those providing some material support to patients, to orphans, vulnerable children we need to be aware of this kind of arrangement that some of the people benefiting could be having depression or other mental health issues. They need to refer them to the facilities.

E: To let them be aware of, mental health issues.

R1: This is important, then also I think indirectly, they will know that, there is something happening because if somebody goes, somehow has depression because of other issues, gets the book but he is also getting the treatment, they will see this person as if he is improving. They will think, they may even think that they are the only ones because they have provided the materials therefore, they have contributed. Yes, true they contributed towards improvement of these patients but then, your efforts, they also need to be appreciated because the anti-depressants plus that relief from lack of materials when combined, the improvement will even be enhanced, that is my thinking.

E: Now … these agents are?

R1: We do, we do through the review meetings.

E: And at that point, if we are sensitizing you people about depression can’t you share with them …?

R1: I think we can, we can share about the programmes that are ongoing in the district.

E: The new one like …

R1: The new ones, so we quarterly meet and conduct a Stake holders Performance review and review meetings, and we talk about the different programmes and achievements. I think we can so that they get to know that there is somebody contributing to that improvement that they are seeing within that patient.

F: So that they also know that part of those others whom … Have that as one of the interventions achieved, then to Health Management level, orientation of these implementing partners. Up you will see (*referring to the ToC map*), it is one of the activities we expect at your level to tell them about this. Any other contribution? Yes, few other comments? I thought we had put in enough fuel. Because that…, are we exhausted? it looks like people are saying they were almost happy with the Map apart from the few adjustments here and there, I thought you would amend it so, so much. Any additions? It looks like we have ran short of ideas. (*Probably meant* *exhausted ideas*)

E: Joshua are you done?

F: I think yeah, we are, we have ran short of ideas.

E: why are you saying we have ran short of ideas, we have got exhausted like this, because we have had three workshops. So I think, let us give a hand-clap to Joshua, our facilitator, I think he has done a great job, getting all these ideas from us and I think he is now going to finalize his map. But this has been a journey which started with a launch and two workshops and this is a third workshop. So I think, let me ask our host madam DHO to say some, give some comments as we come to the end of this exercise.

R1: Thank you and good afternoon everybody? I want to say that, thank you for coming to this meeting. I am sorry I had a radio program somewhere so I was not here right from the beginning, but I want to welcome the team, Professor and the team and also say, thank you for coming and also share with us these now well arranged interventions, because we were here and remember we were here in two groups and we were brain storming on what can happen. The team went back and they have come back with an organized, an organized what is it? Is it a matrix or whatever?

E: A map, map.

R1: It is a map, with an organized map giving the directions to interventions that we are going to implement at the different levels, the community, the health facility and the district level. Every one of us has a role to play, isn’t it so? But we all have a common goal – how can we improve on the management of depression in HIV/ AIDs? That is what we are all aiming at. I want to say thank you for the participation. I am sure more interventions have come up and they are going to be captured so that next time we shall also see them. I understand there is another meeting, but as we think of these interventions, it is very important we know we are a pilot district and, however, we must also benefit and sustain what we have benefited. So some of the interventions should be pointing to how we shall sustain that good management of depression into HIV, within the community, within the house hold. Have we brought out all the interventions that we think can lead to improvement of depression even at the house hold level? And the moment the person is fully recovered from depression, how do we sustain it at the house hold level? And how do we bring in the other members of the house hold to properly manage? Because we know people down there, I know people may be somebody with depression, they do not tie him or her but that is how they are managing. I hope these interventions will help in the improvement of the management at the house hold level, the same to the health facility level. I know most of these case we have been missing them simply because some of our health workers have forgotten, how do you diagnose depression and in case depression has been diagnosed, how do I handle? How do I manage? How do I help this person so that he/she recovers much faster? During the supervision we are seeing antidepressants expiring at the health facilities. We collect them, we bring them into the stores, and nobody asks for them and at the end of the day, they expire. And I hope as we go through this sensitization, those antidepressants are going to be used more and more and also at the district level, I want to promise that we are going to pick a lot of interest. And how many cases we are capturing with depression; I hope the HMIS do…depression, I don’t remember.

E: But it does,

R1: It does, yes, I think this is one of the things that we are going to monitor. How many are entering into the system and I hope also at the national level, they will also realize the magnitude of the problem through the analysis of the data that we shall be sending. I am sure you have heard this with hypertension and diabetes, and one of the things that has been done now in the new clinical guide lines of 2016, I think at the various levels, they have put drugs for DM and hypertension, but whether NMS had delivered them to you, I think that is a different question. So, I have all the hopes that management of depression is going to improve. It is just unfortunate that not all the health facilities are going to benefit directly but I am sure within the re-view meeting somehow, people who are going to become expert in managing depression shall be communicating or sharing their experiences with the other health facilities so that the benefits spread throughout the districts and at the end of the day, we all benefit. I want to say thank you, by the time I left, very few people were here, I want to say thank you to those who kept the time, but I am happy eventually you all turned up. I think with those few remarks, I want to invite the CAO to officially close this meeting. Thank you very much Professor and the team.

R2: Our facilitators and participants, I was also beeping because of the too much work I had outside there, but I want to thank the professor from the launch up to today, from behind the scenes I have been following what is happening and the structuring of the map as doctor has said, it is giving a clear direction of where you were, we have come from, what interventions we are setting, what will be the impact and you are even looking at what will be the outcome. This is something very great. I have looked at the interventions, from what I saw from you getting exhausted, I have quite fully confirmed that these are the issues we need. To pick out some few of the issues that are happening under this project is going to actually help us in other operations that we normally do – the capacity building at health facility level and district level is quite impressive. There was one thing that was emphasized, they were calling district support – that support is there from the beginning up to the end. Now, in Uganda, people are stressed and depressed due to many things, very many things – politically, socially, economically and everything including the life style – drugs; for example the whites *(Bazungu*) who died recently in Kampala, on checking their bodies (*probably referred to postmortem*) they were are full of drugs. And the depression in HIV/AIDs will also help us to tackle other, other depressions that are elsewhere. It can even be in malaria, we do not know. But also what I am not very sure of, is like we integrated TB treatment into the HIV /AIDs management, do we have a policy Professor?

E: A policy on integration?

R2: Yeah,

E: Yes, we have the new Guidelines.

R2: Ok, then that is better. Then once there is a guideline, once there is a policy for us as civil servants, that is an order and that is how we are going to move without even reasoning how it does. As a local government, we are taking HIV operation very seriously. Yesterday we were meeting our partner who is implementing comprehensive HIV care. We would not want any gaps – we want a very clear programme that is going to address all those challenges. You remember few years ago, the virus prevalence was going high and we do not want those gaps, and impressively, the new partner has picked on very well, I can see progressively, the number of people tested for HIV increasingly getting high and we want to keep so closely to them such that the programme is well managed. For my colleagues the civil servants, this is our business (*probably referred to responsibility*). To us this is not a project, this survey is learning for us to improve service. Let us support the processes that are under the survey for the good of our country. You remember our district is piloting the study and the good outcome of the project will benefit the greater Uganda. For God and my country.

E: Thank you very much for those remarks, thank you very much for having accepted us in the district and I think as a research team, we have felt, every time we come here, we have been feeling at home and we hope that things are going to continue going on because, I mean, we still have a number of activities to undertake, although now we have designed the map. I think we now need to look at the therapy itself, so in not a very long time, may be towards the end of March, we shall be inviting, we shall actually be coming back here with an expert from India to discuss, to look at the intervention – the brief psychotherapy, the treatments, what treatments should we put in that package, so please when we call upon you again, accept our invitation and please come and we discuss. Ok, I think with those words, we are going to get a hand shake from Richard Mpango and then we shall go and have our lunch, and then we call it a day. I wish you a good weekend, God bless you.

ENG
