# Supplementary material for: Using Theory of Change to inform the design of the HIV+D intervention for integrating the management of depression in routine HIV care in Uganda
Source: PLoS One. 2021 Nov 30;16(11):e0259425. doi: 10.1371/journal.pone.0259425 (PMC8631669; doi:10.1371/journal.pone.0259425)
Supplement: Attachment — Submitted filename: Response to Reviewers.docx [file pone.0259425.s005.docx]

Dear Reviewers,

We thank you for the useful comments. We have revised the manuscript and addressed the reviewers’ concerns, as highlighted in the revised manuscript (in track changes).

We have also revised the reference list accordingly; making it complete and correct, in line with the submission guidelines (Vancouver style).

We have addressed the additional requirements relating to the formatting. The reviewers’ comments are all addressed, as highlighted below:

Reviewer #1: This is an interesting manuscript that describes how the Theory of Change was used to build consensus, identify key challenges in programmatic development and service delivery, and to develop a consensus plan for potential implementation of programs to treat comorbid depression in PLHIV. The manuscript is well-written, and the authors appropriately dedicate a considerable amount of space describing the Theory of Change as a process for developing strategies to address public health problems. Mental health is a particularly difficult area to work on in LMICs, in light of the lack of dedicated personnel in most contexts, so this process was particularly helpful in highlighting the problem to key stakeholders and building a plant to address it. The manuscript is well-written.

I have only one major comment with regard to the Results section - which could have meaningful implications for re-writing that section and making it more robust. The authors have noted that the TOC focus groups that were conducted with stakeholders were audio-recorded and that transcripts were created of these recordings. However, only one representative quotation is included in the results section on page 17 (describing challenges in detecting depression in these patients).

In comparison to the rest of the paper, the Results section seems rather thin, and it would benefit from better presentation of empirical data that informed the TOC map / figure, which is the main finding of their research process. However, all of the critical points in this TOC map were presumably informed by actual statements and feedback provided in the stakeholder meetings / focus groups. The authors should strongly consider including selective representative quotations on key points raised by stakeholders, with regard to their concerns about resources limitations, the need for higher level buy-in and resource provision, challenges raised by community stakeholders, etc. This will not only make the paper more rich, but it will help empirically justify and clarify what is in the TOC map - which otherwise seems to arise from thin air.

Obviously, there are limited quotations that can be introduced, but at least 5 or 6 should be considered to flesh out key points in the TOC map.

*This has been addressed. We have added some selective representative quotes on key points raised by stakeholders in the results section*

Reviewer #2: Well done Authors.

Please find areas to be attended to below.

Abstract
Method: Please name the district, region and country where the study was conducted.
Please maintain either PLWH or PLWHA in all write-up instead of interchanging them.

*This has been addressed. The name of district, region and country have been added. We are also maintaining PLWHA throughout the manuscript.*

Background
PLWH prevalence in Uganda and if possible the region and district of study site? This should appear in paragraph 1. The rates in Uganda/region/district of study may be lower than the global and sub-Saharan but these rates could be increasing or not changing over a range of years and this may also be a concern I think. What about the association with depression in Uganda, region and district of study site?

*Information on the estimated prevalence of PLWHA in Uganda and the central region (where the study site is found) has been provided in paragraph 1 as suggested. Reliable information on the prevalence in study district in particular is not readily available.*

*We have also included a statement on the association between HIV and depression in Uganda in paragraph 1.*

Methods
When was this study conducted?

*We have indicated in the manuscript when this component of the study was conducted (between November 2017 and February 2018).*

How did you take care of anonymity and confidentiality?
*The ToC workshop proceeding (for the 3 workshops) were all audio-recorded and later transcribed. During the transcribing, we did not include any potential identifiers, not even initials. We were simply interested in capturing participants’ views, and were all represented with “P” in the transcriptions.*

Ethical
Ethics clearance number for UNCST not quoted.

*The ethics clearance number has been quoted.*

Data Analysis in 2 sections?
Please merge the different sections to have only one on data analysis. The first section seems hanging.
The merging should be well labelled as 2 different sections namely:
Data Collection
Data Analysis

*This has been done.*

Work Published elsewhere?
This article should be referenced so that readers can refer to it. In case it is not yet published, then the reader will be disadvantaged.

*This work (manuscript) is submitted to PLOS ONE journal, and has not been published elsewhere.*

Results
The study had a number of individual and group discussions but in this section, only one conversation has been quoted? Each subsection (of impact, outcomes, interventions, indicators) should be accompanied by at least 1 conversation, otherwise if left plain as it is makes the reader think the authors have written their perceptions.

*We have added more quotes in the results section*.

Discussion
Are there any of your findings which other studies did find too?
What new knowledge has your study added to the field of research and science?

*A related study looked at use of ToC to develop a brief psychological intervention for common mental disorders (not depression specifically); and found the approach quite useful.*

*The study generates knowledge on the practicality of how to integrate the management of depression in HIV care, as highlighted.*

Conclusion
This seems so thin. Of what importance are the study findings to individual patients, health decision makers and implementers, Uganda and the globe if any?

*The conclusion has been revised, making some addition*

Authors’ Contribution
RM is the project lead or RM as the project lead…... Can one of words “the” be deleted so the statement end as …in the study design?

*This has been revised.*

References
Can references with internet links be accompanied with when this was accessed?
There is need for edits to be done on the references. For some, the journal is in italics and others not. Is the journal name and year of publication separated by a dot or not? Is year, journal number and pages supposed to have spaces or not? There has to be uniformity.

*The reference list has been fully revised, in line with the journal’s guidelines (Vancouver style) and addressing the above concerns.*

Once again, we thank you for the useful comments that have helped us produce a better version of manuscript.

Yours Sincerely,

Joshua Ssebunnya

(Corresponding author)
