# Supplementary material for: Using Theory of Change to inform the design of the HIV+D intervention for integrating the management of depression in routine HIV care in Uganda
Source: PLoS One. 2021 Nov 30;16(11):e0259425. doi: 10.1371/journal.pone.0259425 (PMC8631669; doi:10.1371/journal.pone.0259425)
Supplement: Attachment — Submitted filename: Response to Reviewer.docx [file pone.0259425.s006.docx]

Dear Editor,

Once again, we thank you for the useful comments towards improving the manuscript. These have been addressed as below:

1. Please review and adhere to the journal's expectation for reporting qualitative research: <https://journals.plos.org/plosone/s/submission-guidelines#loc-qualitative-research>

We have adhered to the journal’s expectations for reporting qualitative research. However, the data collection methods for the study being reported were not the usual qualitative methods (such as interviews or focus group discussions), but rather workshop proceeding. We have endeavored to include the aspects commonly reported under the methods section.

2. There is very limited information given around the data analysis. No references are provided for content analysis. The subsequent interviews and analysis which proceeds as part of another phase of this study need not be mentioned here at all. However far greater information about the analysis process for this set of workshops is needed. Simply stating content analysis is in adequate. Who analyzed the data, were software used, how was rigour facilitated, how was the ToC used to guide analysis / themes, etc. (Completing a checklist such as COREQ may assist the team in building up their analysis section.)

We have provided more information on data analysis and left out information on the subsequent interviews and analysis that followed, in another phase. A COREQ checklist has also been completed for this purpose and sent as an additional file.

3. The supplementary files are raw data, including a lot of information about participants, especially page 1 of each supplementary file (and upon reading some of the transcripts, potentially identifiable data about the participants, by way of their descriptions and stories). Whether this level of sharing outside of the study team was included in the consent form is not clear. So the authors should either share the consent form to clarify what exactly participants agreed to, or then please remove all supplementary transcripts and state that de-identified data will be made available upon request, but the full data set may not be shared due to the qualitative and potentially identifiable nature of the raw data (eg, transcripts).

*We have removed the supplementary files (transcripts) as advised, because the nature of the data doesn’t allow anonymity. The consent forms didn’t allow this level of sharing outside the study team. We have further stated that the de-identified data will be made available upon request.*

4. Similarly, Table 1 must be removed as it may be too revealing. I gather there are only a few people holding some of these positions in Mpigi district and it is unclear if their privacy could be breached. If this is not the case, then the authors may retain Table 1 but make it clear in the text that many people hold these positions, to ensure no one participant's identity is inadvertently breached (eg, MH specialist, Asst Chief Admin Officer). I suggest stating that 1) 3 workshops were held with a range of relevant stakeholders - including the various stakeholder/participant types (add total N and n per workshop), and 2) that workshops included people working in similar job ranks to enable open dialogue, etc. If there is anything further the viewer should know about the workshops, for example, key differences between the three workshops in terms of questions asked/data collected or the way the interviews went, then please share that as well. It is for example unclear if the same people attended more than one workshop or all participants were unique. If same people attended >1 workshop , then we need to understand exactly what the differences between the workshops were (eg, in terms of questions asked).

*We are retaining table 1. It has been revised to avoid mentioning positions that are held by few people in the district; and is not too revealing in its current state. We have made it clear that there are quite a number of people in each of the groups/categories mentioned, and the privacy of those that participated cannot be breached.*

*It is also made clear that workshop 1 and workshop 2 were attended by different people, and followed the same agenda/procedure. Workshop 3, was largely for consensus building after reviewing the draft ToC map and brought together stakeholders who had attended the first 2 workshops. The differences in the 3 workshops is indicated in the text.*
